# Supplementary figures and images for: Structures of G-protein coupled receptor HCAR3 in complex with selective agonists reveal the basis for ligand recognition and selectivity
Source: PLoS Biol. 2025 Dec 8;23(12):e3003480. doi: 10.1371/journal.pbio.3003480 (PMC12685177; doi:10.1371/journal.pbio.3003480)

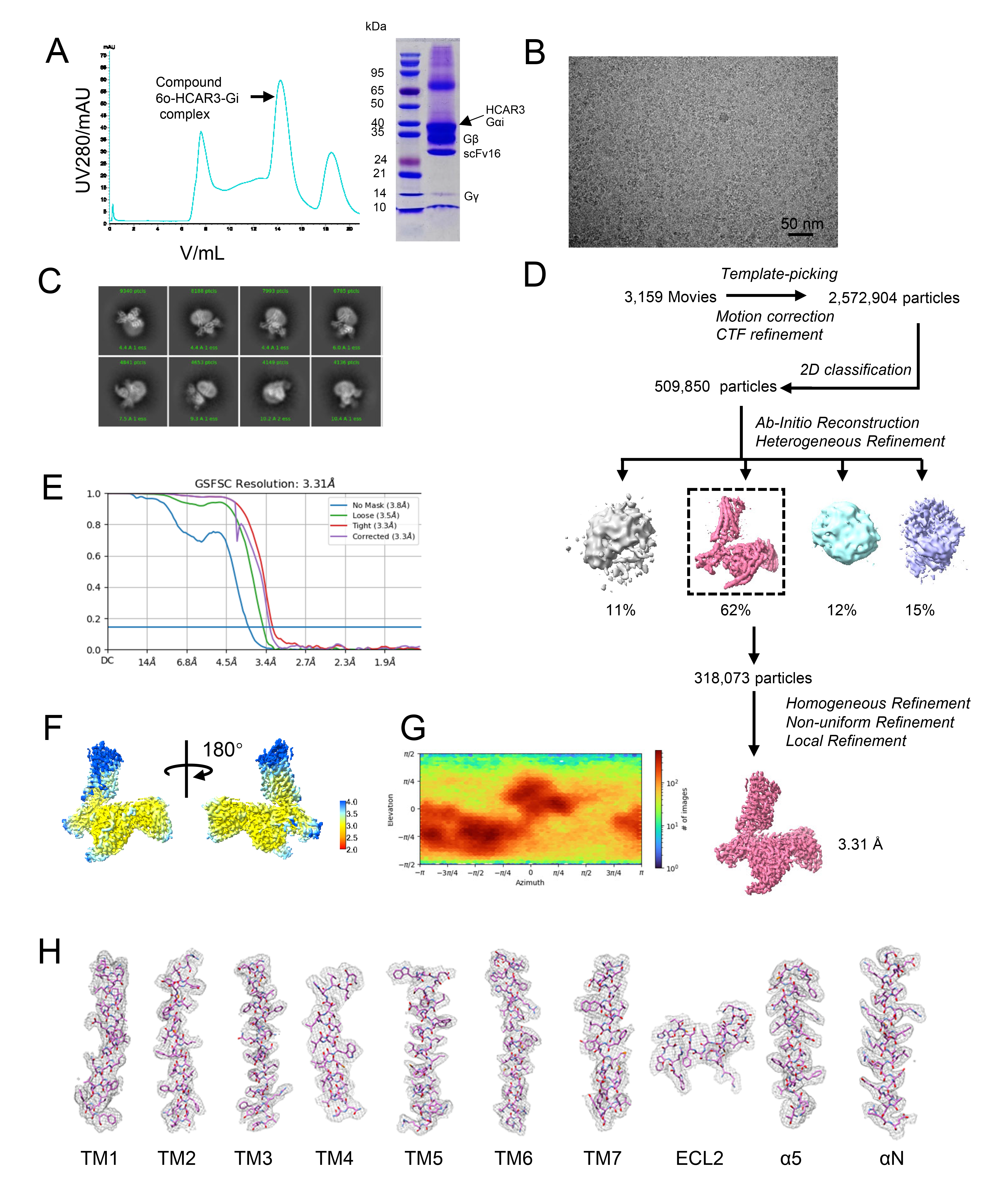

Supplement: S1 Fig — (A) Size exclusion chromatography profile and SDS-PAGE of the HCAR3-Gi1 complex bound to compound 6O. (B) Representative micrograph of the complex particles. (C) Representative 2D averages. (D) Workflow for cryo-EM image processing. (E) Gold-standard FSC curves of the 3D reconstructions. (F) Local resolution map of the complex. (G) Angular distribution calculated in cryoSPARC for the final 3D reconstruction of 6O-HCAR3-Gi1 complex. (H) Representative density maps and models (Contour level 4.20 rmsd) for TM1–7 and ECL2 of HCAR3 as well as the α helices of Gαi1 (αN and α5). (TIF) [file pbio.3003480.s001.tif]

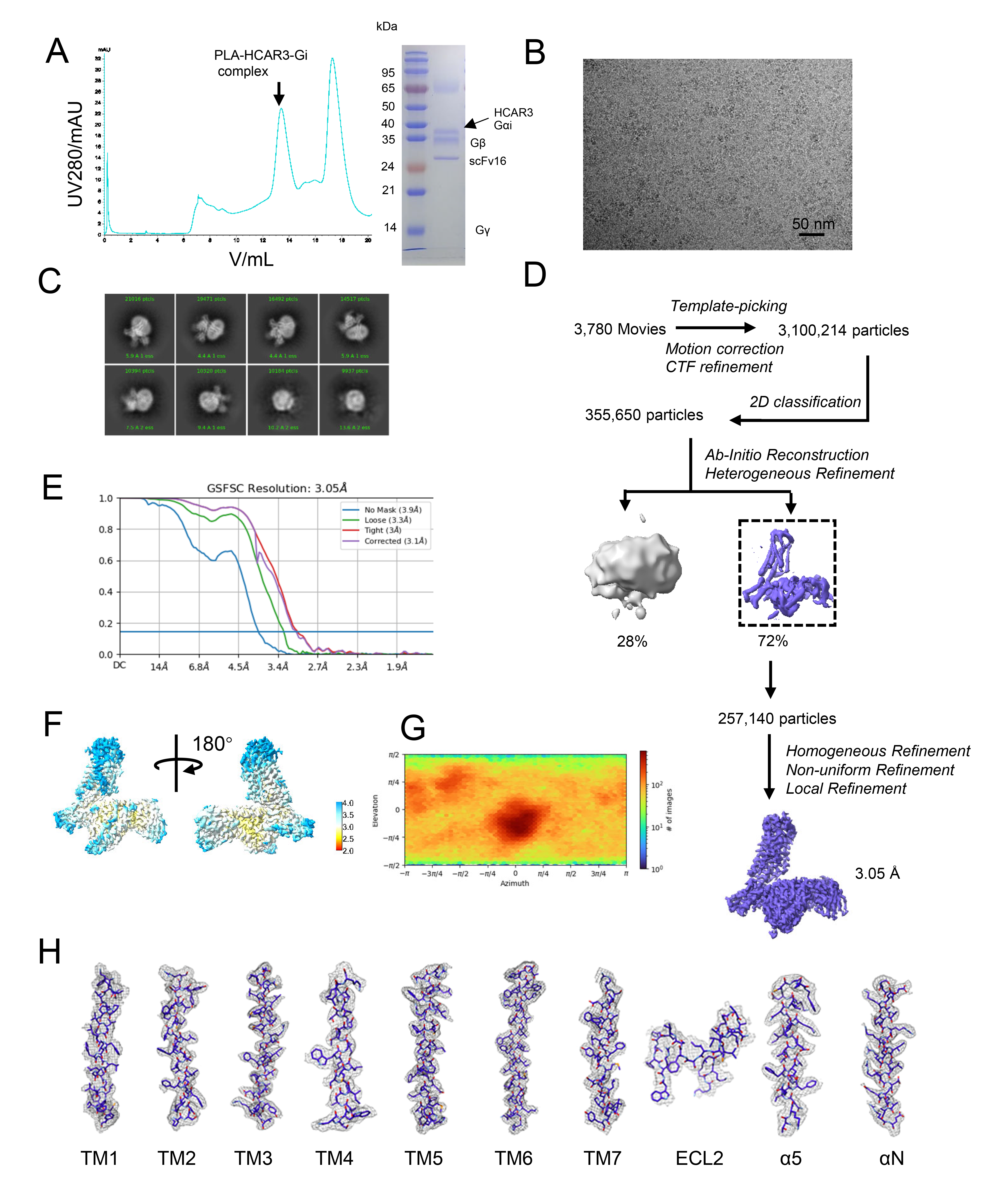

Supplement: S2 Fig — (A) Size exclusion chromatography profile and SDS-PAGE of the HCAR3-Gi1 complex bound to PLA. (B) Representative micrograph of the complex particles. (C) Representative 2D averages. (D) Workflow for cryo-EM image processing. (E) Gold-standard FSC curves of the 3D reconstructions. (F) Local resolution map of the complex. (G) Angular distribution calculated in cryoSPARC for the final 3D reconstruction of PLA-HCAR3-Gi1 complex. (H) Representative density maps and models (Contour level 5.60 rmsd) for TM1–7 and ECL2 of HCAR3 as well as the α helices of Gαi1 (αN and α5). (TIF) [file pbio.3003480.s002.tif]

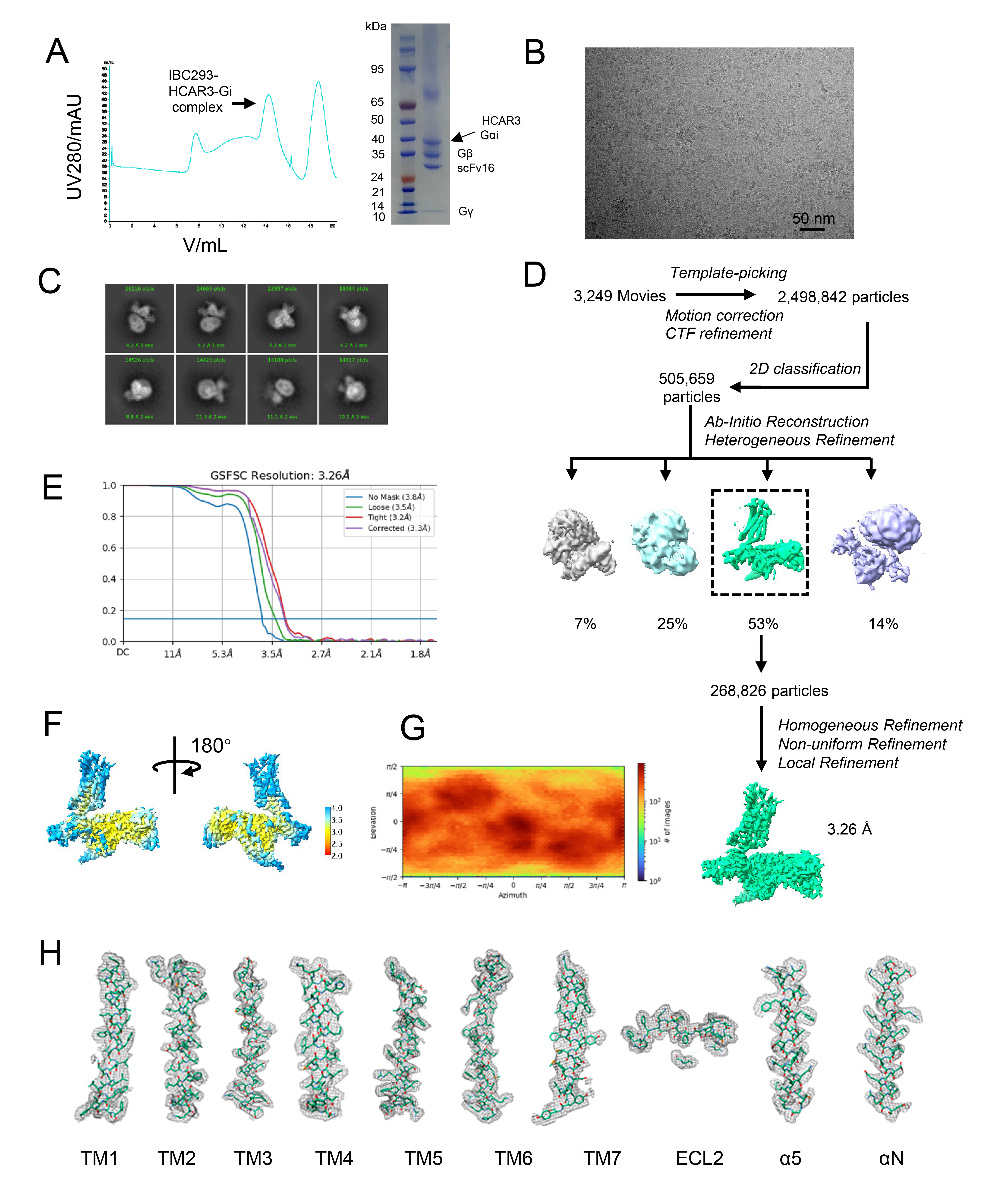

Supplement: S3 Fig — (A) Size exclusion chromatography profile and SDS-PAGE of the HCAR3-Gi1 complex bound to IBC293. (B) Representative micrograph of the complex particles. (C) Representative 2D averages. (D) Workflow for cryo-EM image processing. (E) Gold-standard FSC curves of the 3D reconstructions. (F) Local resolution map of the complex. (G) Angular distribution calculated in cryoSPARC for the final 3D reconstruction of IBC293-HCAR3-Gi1 complex. (H) Representative density maps and models (Contour level 3.90 rmsd) for TM1–7 and ECL2 of HCAR3 as well as the α helices of Gαi1 (αN and α5). (TIF) [file pbio.3003480.s003.tif]

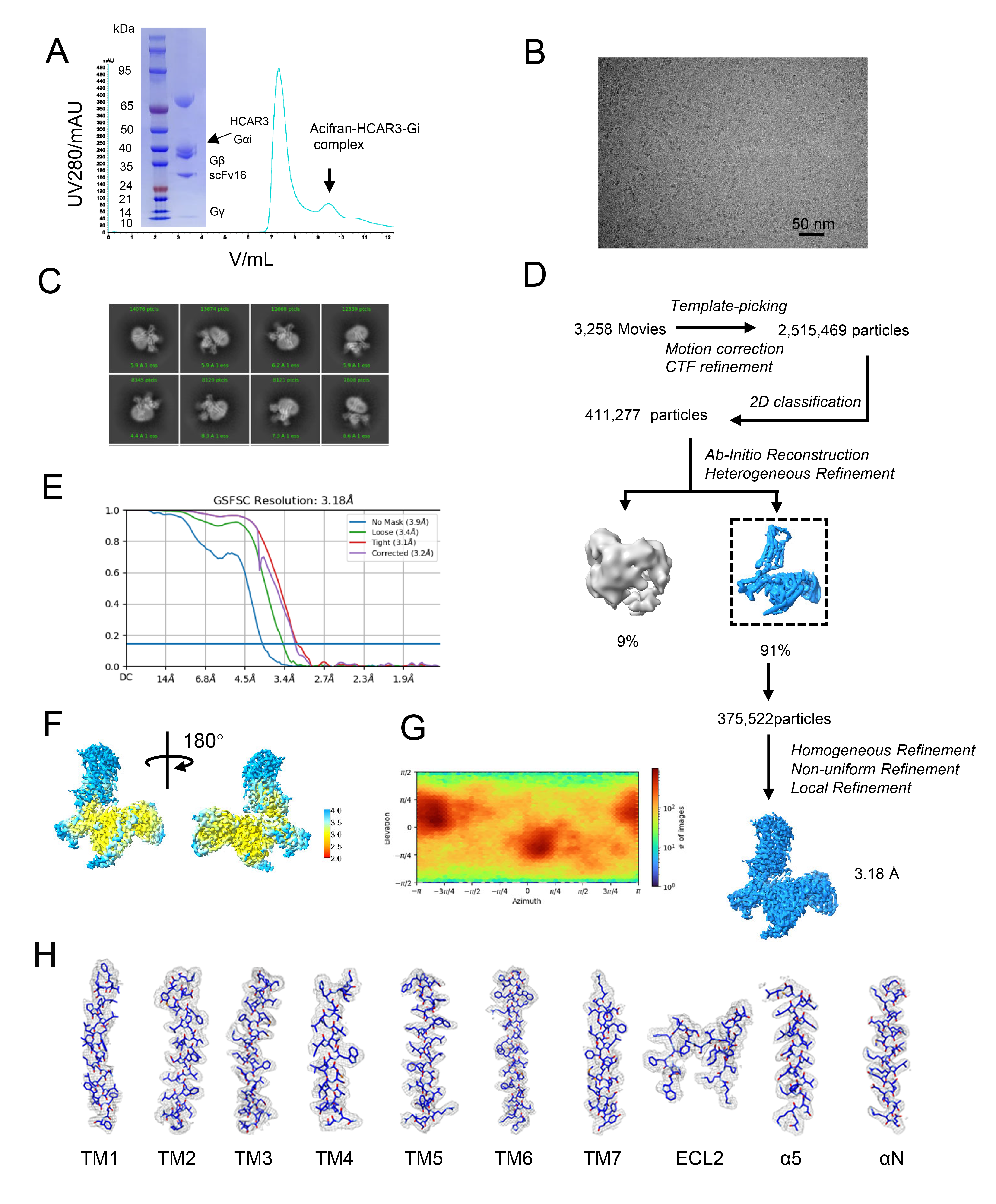

Supplement: S4 Fig — (A) Size exclusion chromatography profile and SDS-PAGE of the HCAR3-Gi1 complex bound to acifran. (B) Representative micrograph of the complex particles. (C) Representative 2D averages. (D) Workflow for cryo-EM image processing. (E)Gold-standard FSC curves of the 3D reconstructions. (F) Local resolution map of the complex. (G) Angular distribution calculated in cryoSPARC for the final 3D reconstruction of Acifran-HCAR3-Gi1 complex. (H) Representative density maps and models (Contour level 4.70 rmsd) for TM1–7 and ECL2 of HCAR3 as well as the α helices of Gαi1 (αN and α5). (TIF) [file pbio.3003480.s004.tif]

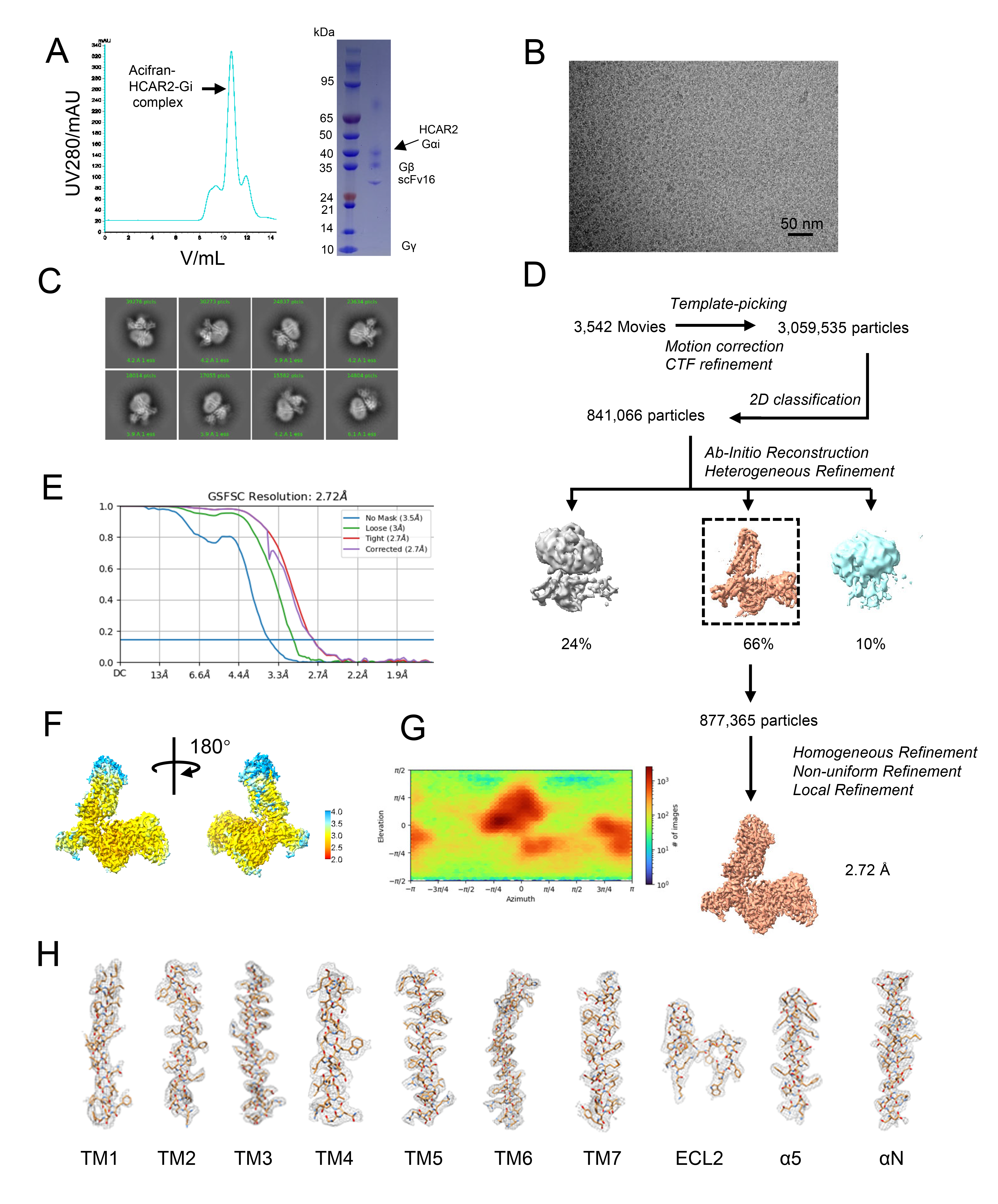

Supplement: S5 Fig — (A) Size exclusion chromatography profile and SDS-PAGE of the HCAR2-Gi1 complex bound to acifran. (B) Representative micrograph of the complex particles. (C) Representative 2D averages. (D) Workflow for cryo-EM image processing. (E) Gold-standard FSC curves of the 3D reconstructions. (F) Local resolution map of the complex. (G) Angular distribution calculated in cryoSPARC for the final 3D reconstruction of Acifran-HCAR2-Gi1 complex. (H) Representative density maps and models (Contour level 4.30 rmsd) for TM1–7 and ECL2 of HCAR2 as well as the α helices of Gαi1 (αN and α5). (TIF) [file pbio.3003480.s005.tif]

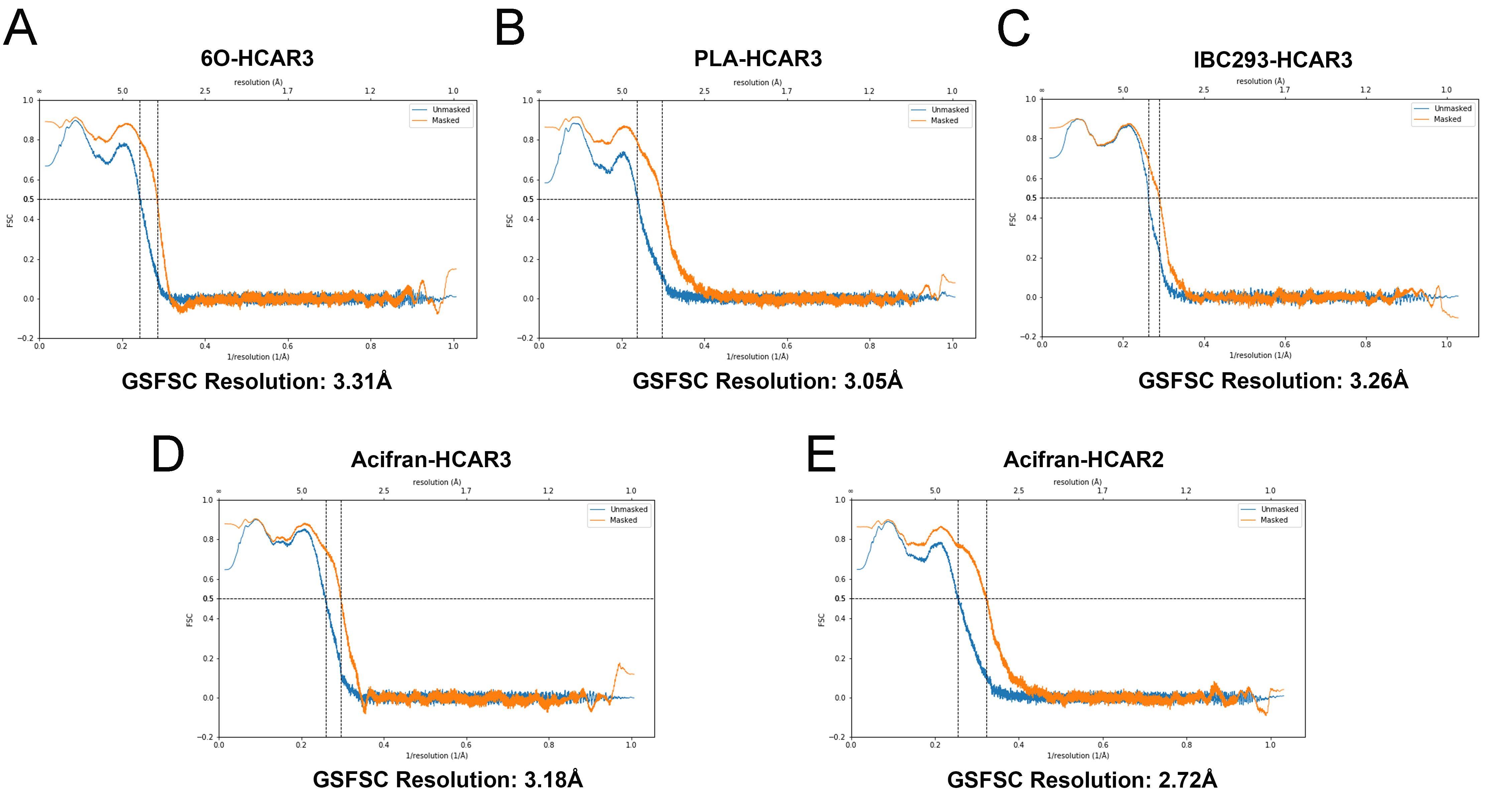

Supplement: S6 Fig — (A) Map to model FSC curve of 6O-HCAR3 complex. (B) Map to model FSC curve of PLA-HCAR3 complex. (C) Map to model FSC curve of IBC293-HCAR3 complex. (D) Map to model FSC curve of Acifran-HCAR3 complex. (E) Map to model FSC curve of Acifran-HCAR2 complex. (TIF) [file pbio.3003480.s006.tif]

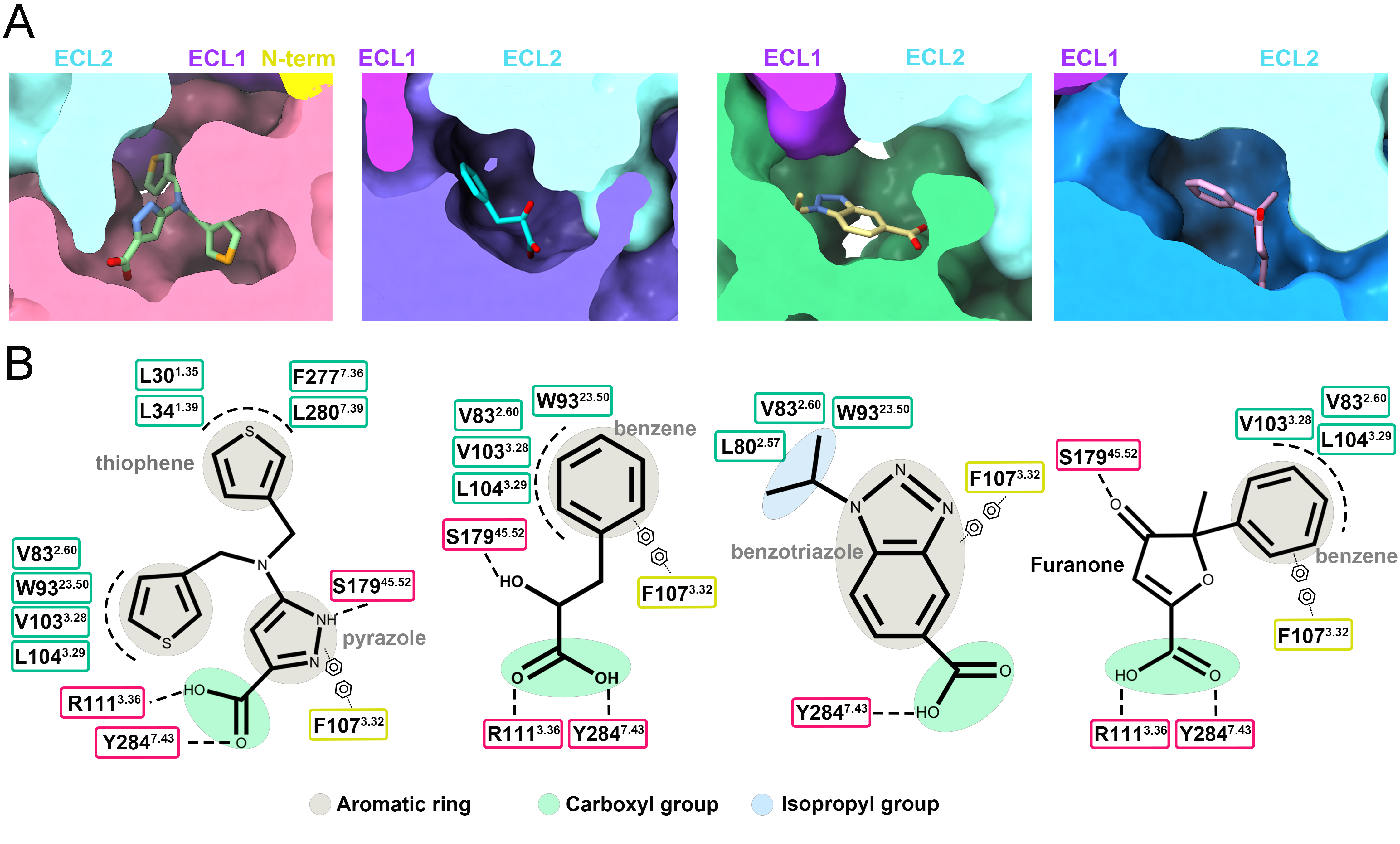

Supplement: S7 Fig — (A) Cross-section of HCAR3 binding pockets in the presence of 6O, PLA, IBC293, and acifran. N-terminal, ECL1, and ECL2 are marked with yellow, purple, and cyan, respectively. (B) 2D presentation of agonist chemical structures and interactions with HCAR3 residues. Residues in pink rectangle: polar interactions (hydrogen bonds, salt bridge). Residues in yellow rectangle: π–π interactions. Residues in green rectangle: hydrophobic interactions. (TIF) [file pbio.3003480.s007.tif]

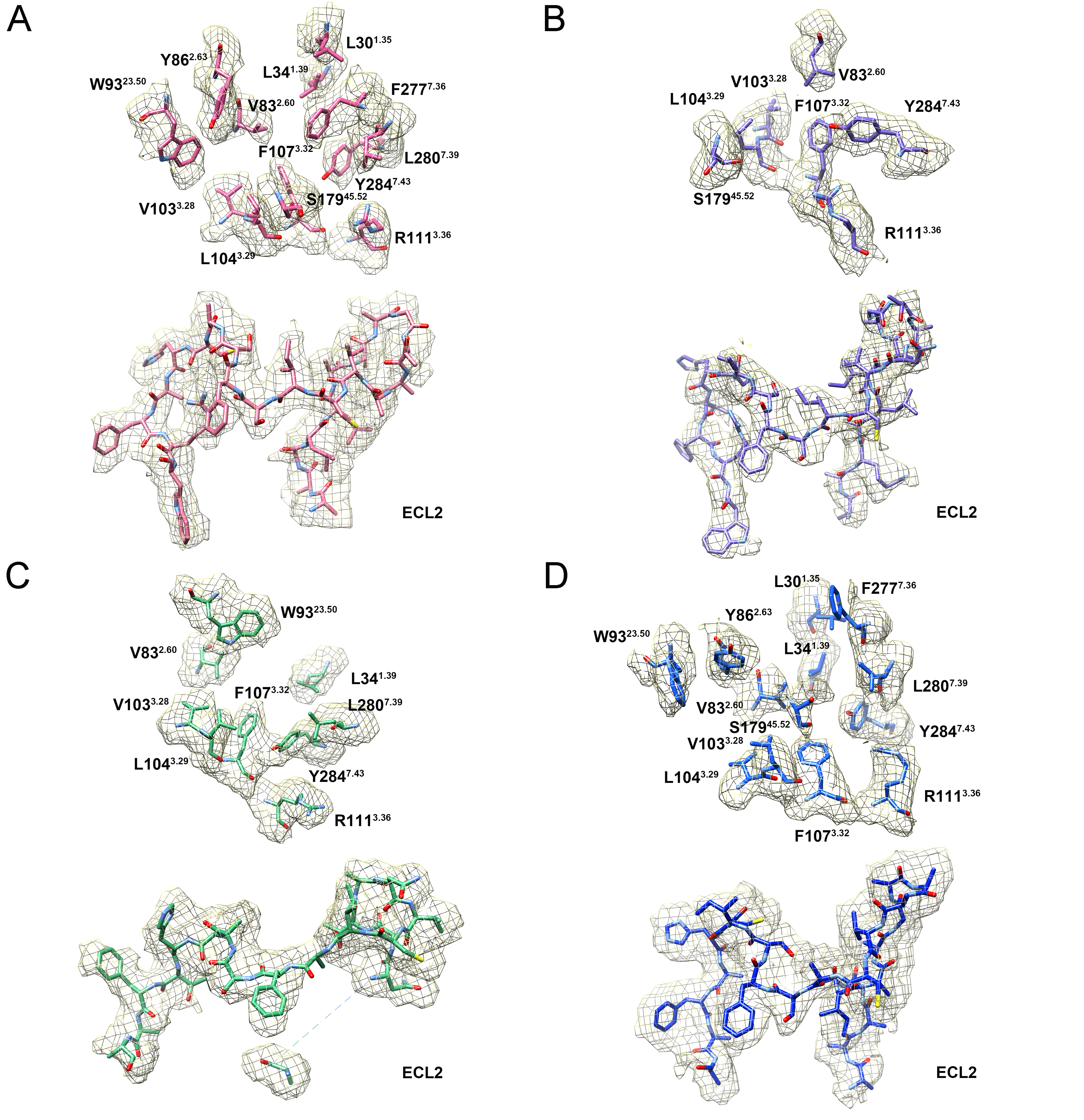

Supplement: S8 Fig — (A) Density of ligand-interacting residues and ECL2 in the 6O-HCAR3 complex (Contour level 6O 4.20 rmsd). (B) Density of ligand-interacting residues and ECL2 in the PLA-HCAR3 complex (Contour level PLA 5.60 rmsd). (C) Density of ligand-interacting residues and ECL2 in the IBC293-HCAR3 complex (Contour level 6O 3.90 rmsd). (D) Density of ligand-interacting residues and ECL2 in the acifran-HCAR3 complex (Contour level acifran 4.70 rmsd). (TIF) [file pbio.3003480.s008.tif]

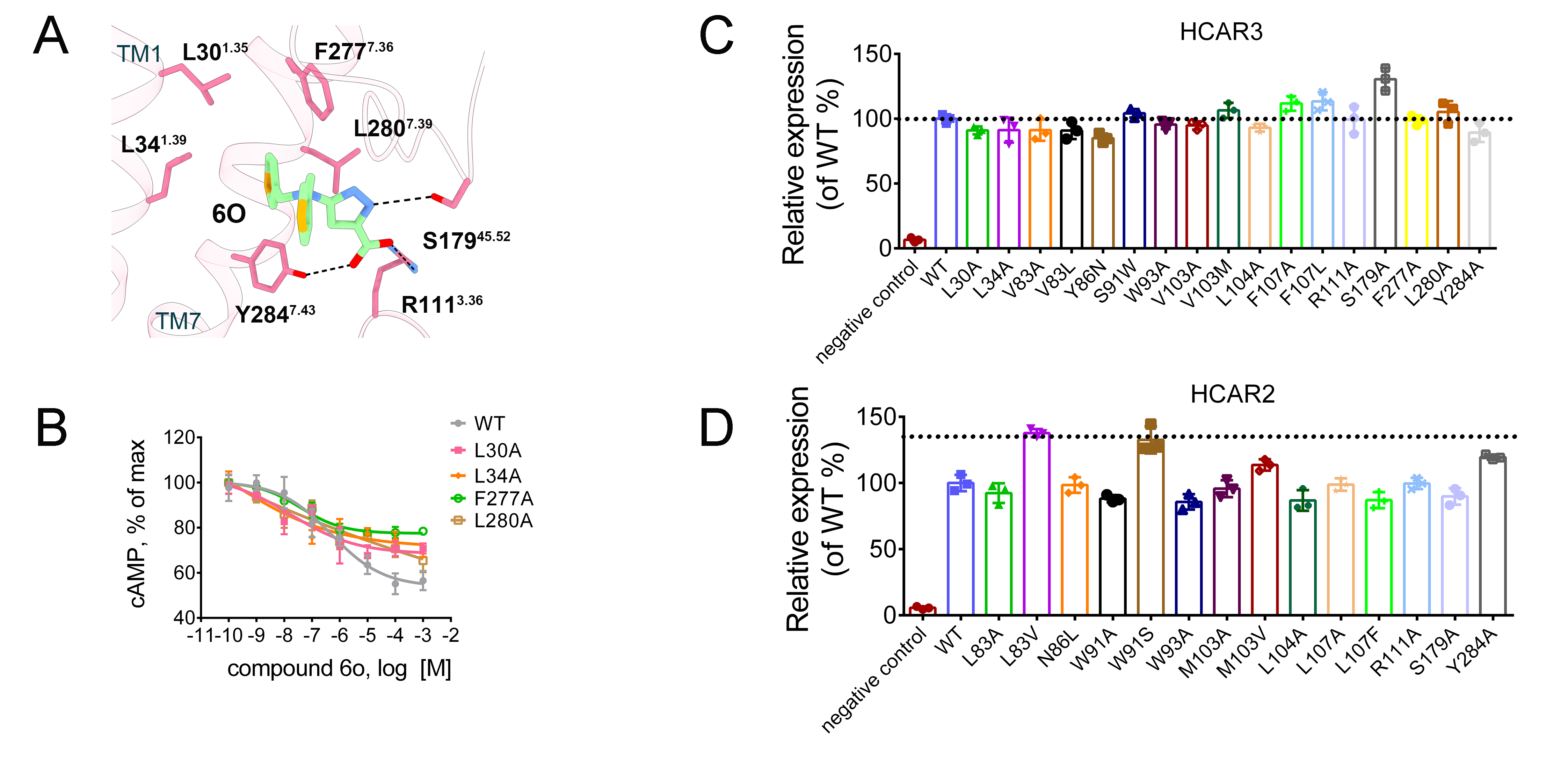

Supplement: S9 Fig — (A) Additional hydrophobic interaction with L301.35, L341.39, F2777.36, and L2807.39 in 6O-bound HCAR3 structure. (B) Effect on Gi-mediated cAMP by single point mutation of several residues that interact with 6O. (C, D) Cell surface expression level of wild-type and single point mutant HCAR3 (C) and HCAR2 (D). The data are presented as means ± SEM. The experiments are performed in triplicates. The underlying data for this figure can be found in S1 Data. (TIF) [file pbio.3003480.s009.tif]

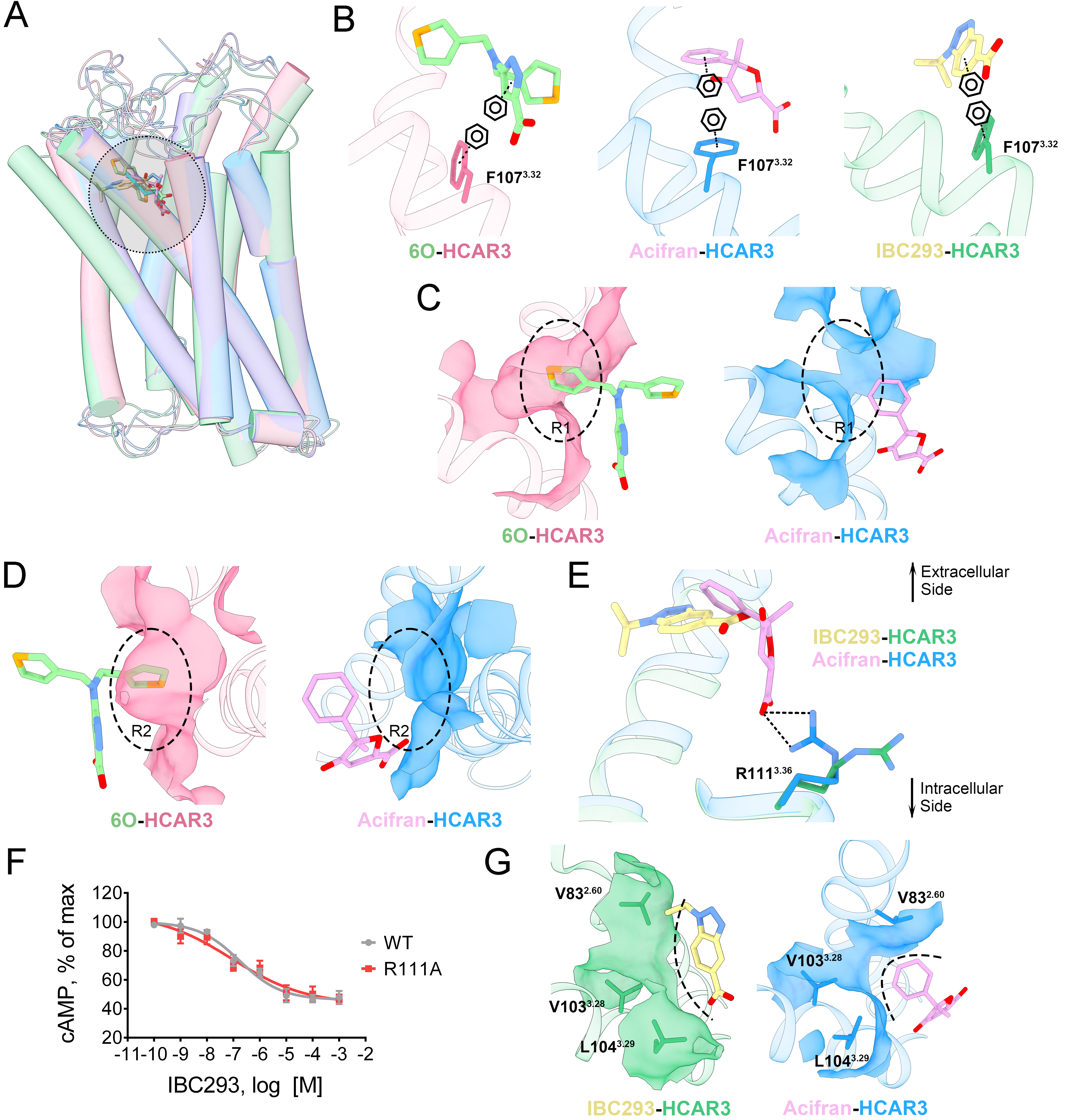

Supplement: S10 Fig — (A) Superposition of 4 HCAR3 complexes aligned based on the receptor regions. The orthosteric binding pockets were highlighted with the dashed circle. (B) π–π interactions between the aromatic moiety of ligands and F1073.32. (C) Surface view of R1 residues from 6O-bound and acifran-bound structures. R1 of acifran-bound structure is obviously looser than R1 of 6O-bound structure. (D) Surface view of R2 residues from 6O-bound and acifran-bound structures. R2 of 6O-bound structure formed a groove-like surface. (E) IBC293 lacked salt bridge interactions with R1113.36 while acifran possesses. (F) Effect on IBC293-induced HCAR3 activation by single point mutation of R1113.36A. (G) Surface view of R1 residues from IBC293-bound and acifran-bound structures. The underlying data for this figure can be found in S1 Data. (TIF) [file pbio.3003480.s010.tif]

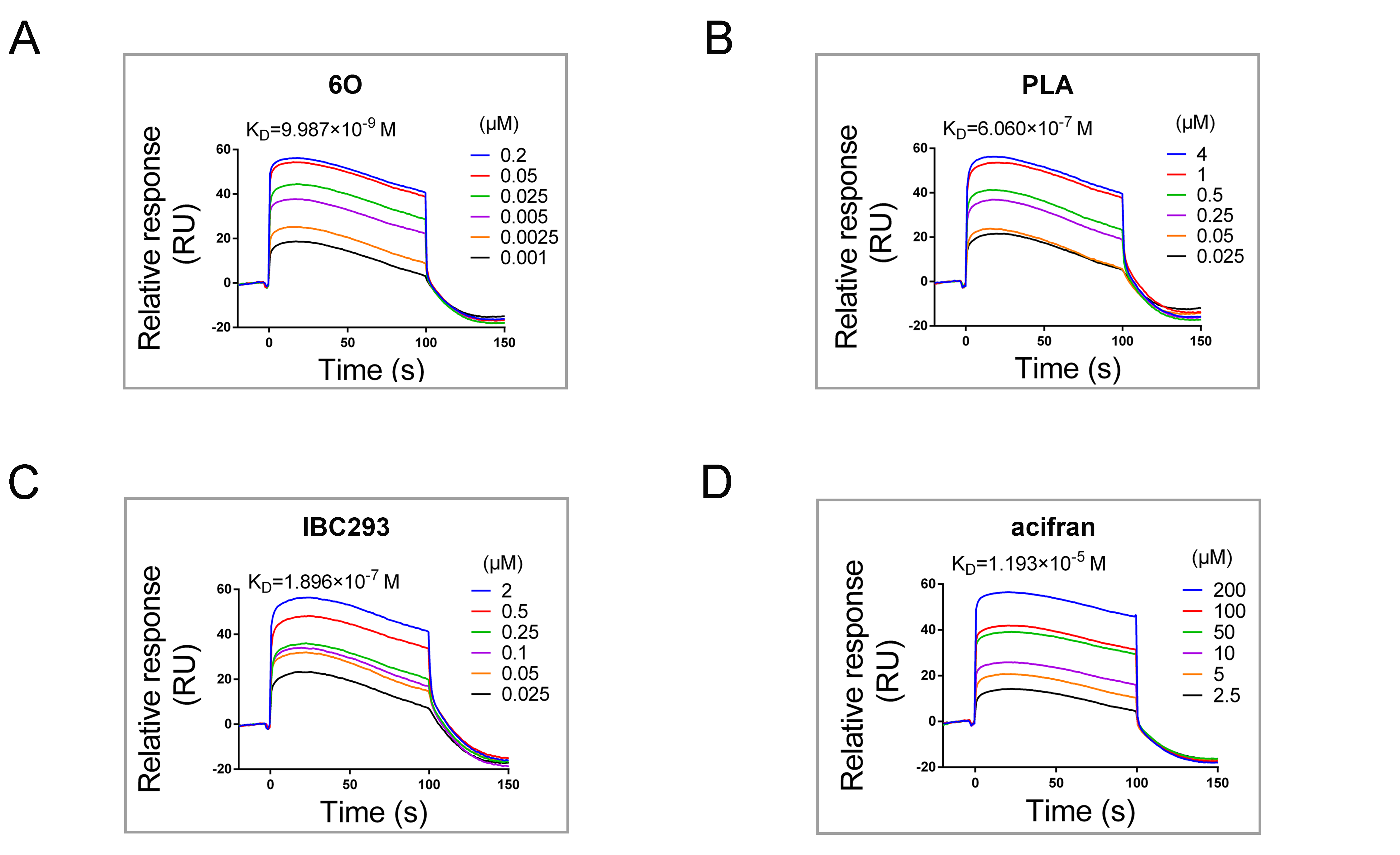

Supplement: S11 Fig — (A) Binding affinity of the wild-type HCAR3 for agonist 6O. (B) Binding affinity of the wild-type HCAR3 for agonist PLA. (C) Binding affinity of the wild-type HCAR3 for agonist IBC293. (D) Binding affinity of the wild-type HCAR3 for agonist acifran. Binding affinity is determined by surface plasmon resonance analysis. The data are presented as means ± SEM. The experiments are performed in triplicates. The underlying data for this figure can be found in S1 Data. (TIF) [file pbio.3003480.s011.tif]

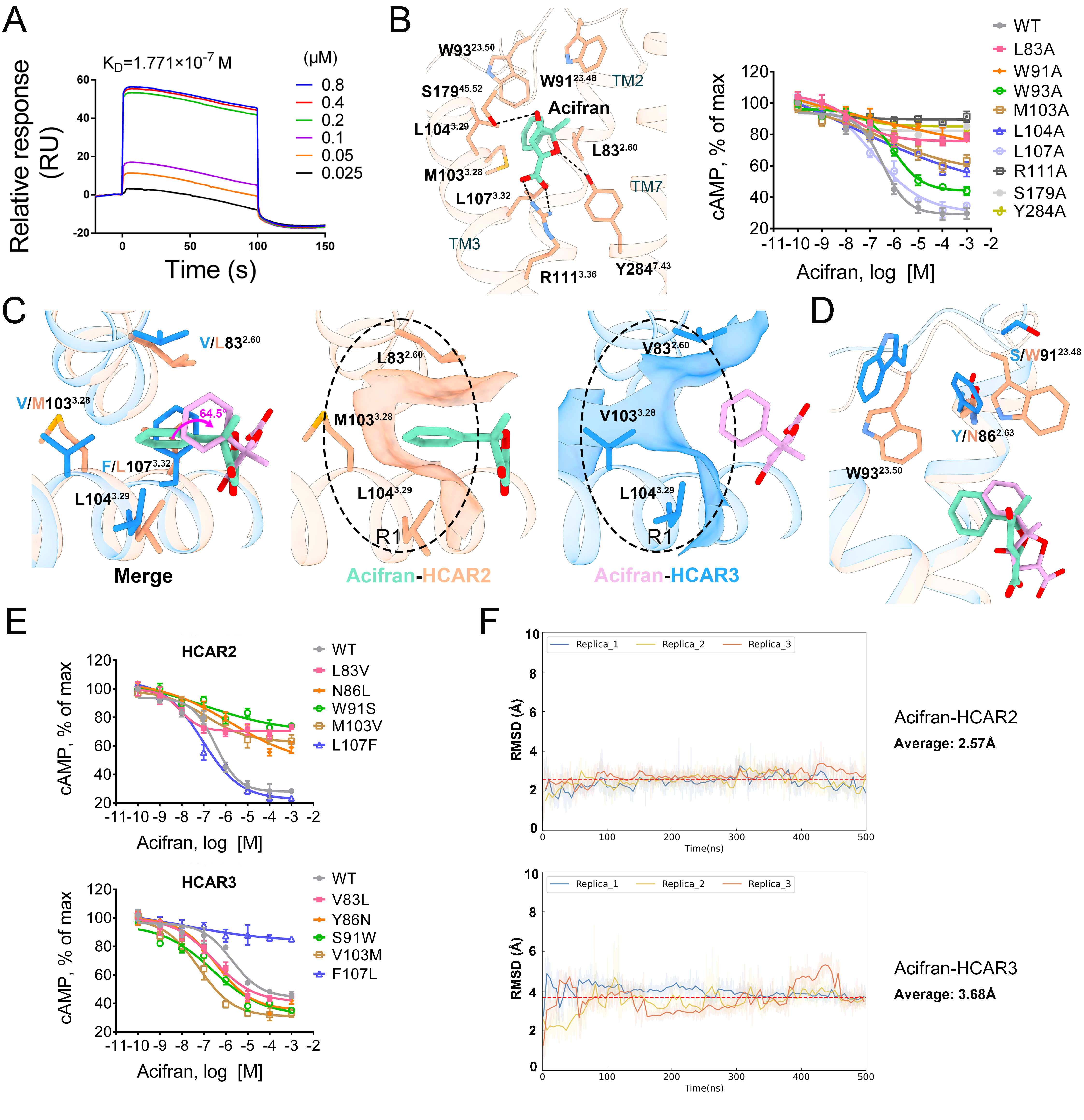

Supplement: S12 Fig — (A) Binding affinity of the wild-type HCAR2 for agonist acifran. (B) HCAR2 key residues interacting with acifran and effects on HCAR2 activation by single point mutation of these residues. (C, D) Detailed comparison of R1 residues between acifran-HCAR2 and acifran-HCAR3 structures. Benzene group of acifran fit R1 better in HCAR2 than in HCAR3. (E) Acifran induced cAMP inhibition of WT HCAR2 and homology-mutated HCAR2, WT HCAR3 and homology-mutated HCAR3. (F) Acifran stability evaluation in HCAR2 and HCAR3. RMSD value Raw (light color) and smoothed (10 ns moving average, dark color). Red dashed line is the average RMSD value for three replica trajectories. The underlying data for this figure can be found in S1 Data. (TIF) [file pbio.3003480.s012.tif]

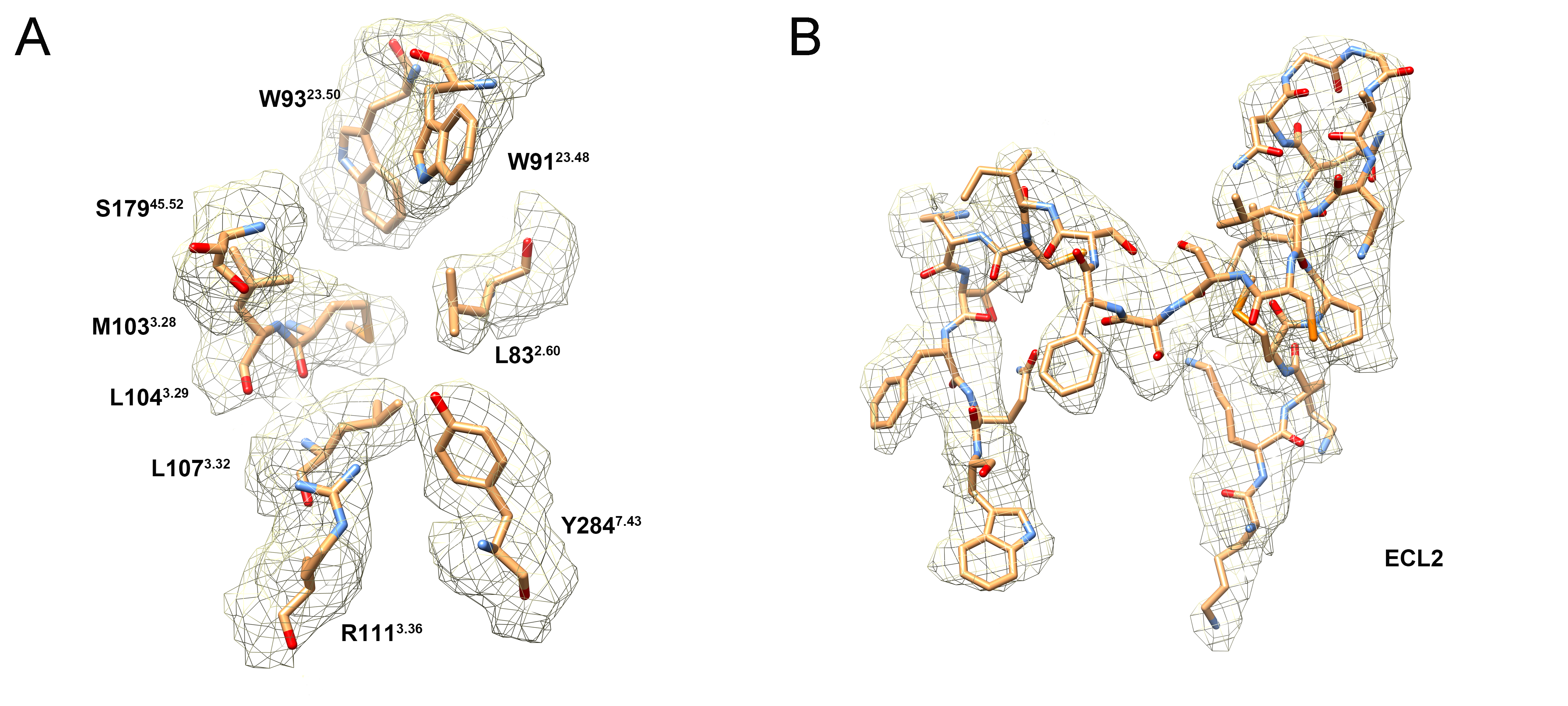

Supplement: S13 Fig — (A) Electron density of polar and hydrophobic residues in HCAR2 that interact with acifran (Contour level 4.30 rmsd). (B) Electron density of ECL2 in HCAR2 (Contour level 4.30 rmsd). (TIF) [file pbio.3003480.s013.tif]

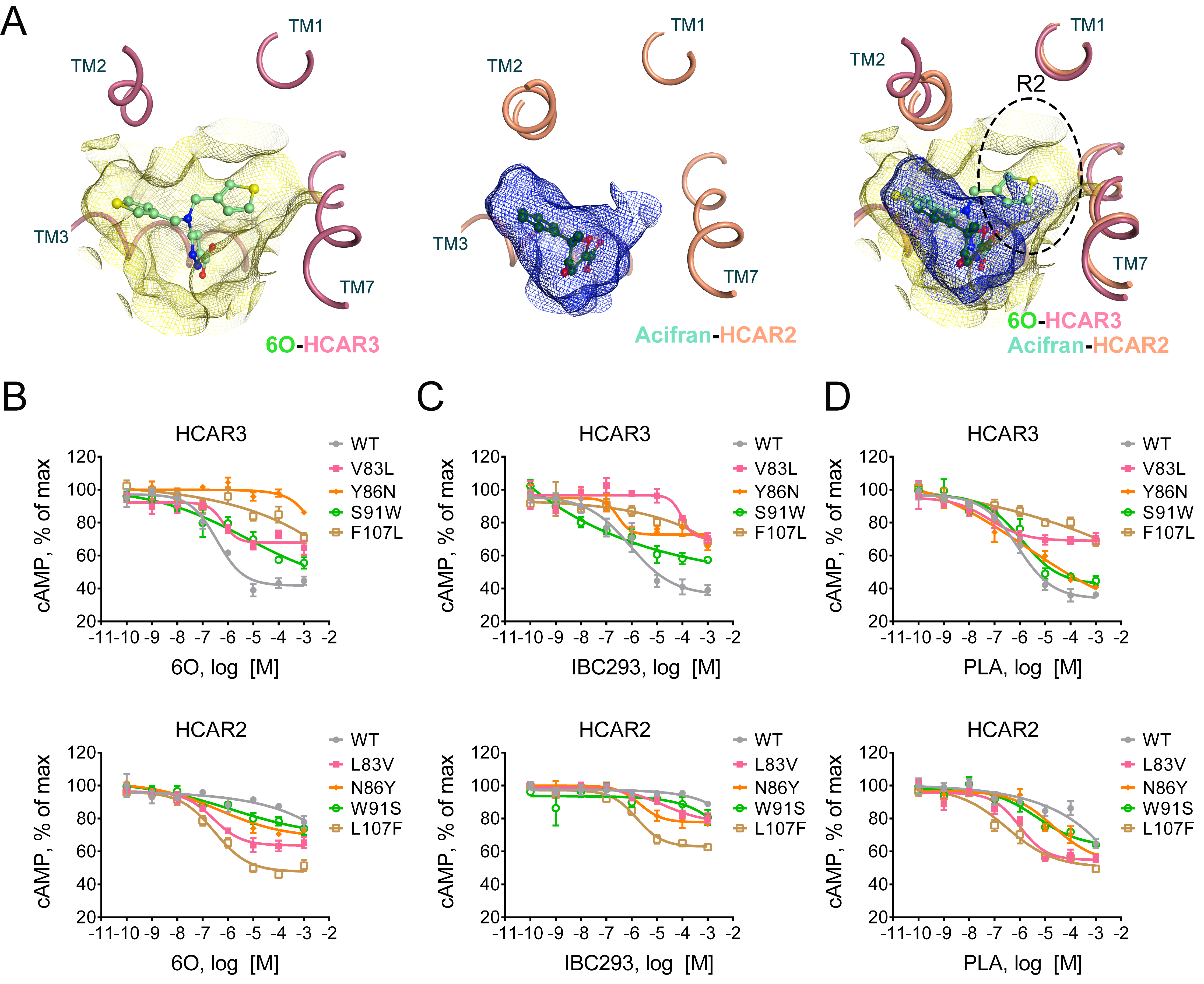

Supplement: S14 Fig — (A) Surface representation of HCAR3 and HCAR2 orthosteric pocket, as well as superimposed HCAR2 and HCAR3 orthosteric pockets. The structures and agonists are colored differently. Medium aquamarine and light salmon, acifran-HCAR2; light green and pale violet red, 6O-HCAR3. Blue, HCAR2 surface; yellow, HCAR3 surface. (B–D) Effects on 6O, IBC293, PLA-induced HCAR3/HCAR2 activation by homology mutation of 832.60, 862.63, 9123.48, and 1073.32. The data are presented as means ± SEM. The experiments are performed in triplicates. The underlying data for this figure can be found in S1 Data. (TIF) [file pbio.3003480.s014.tif]

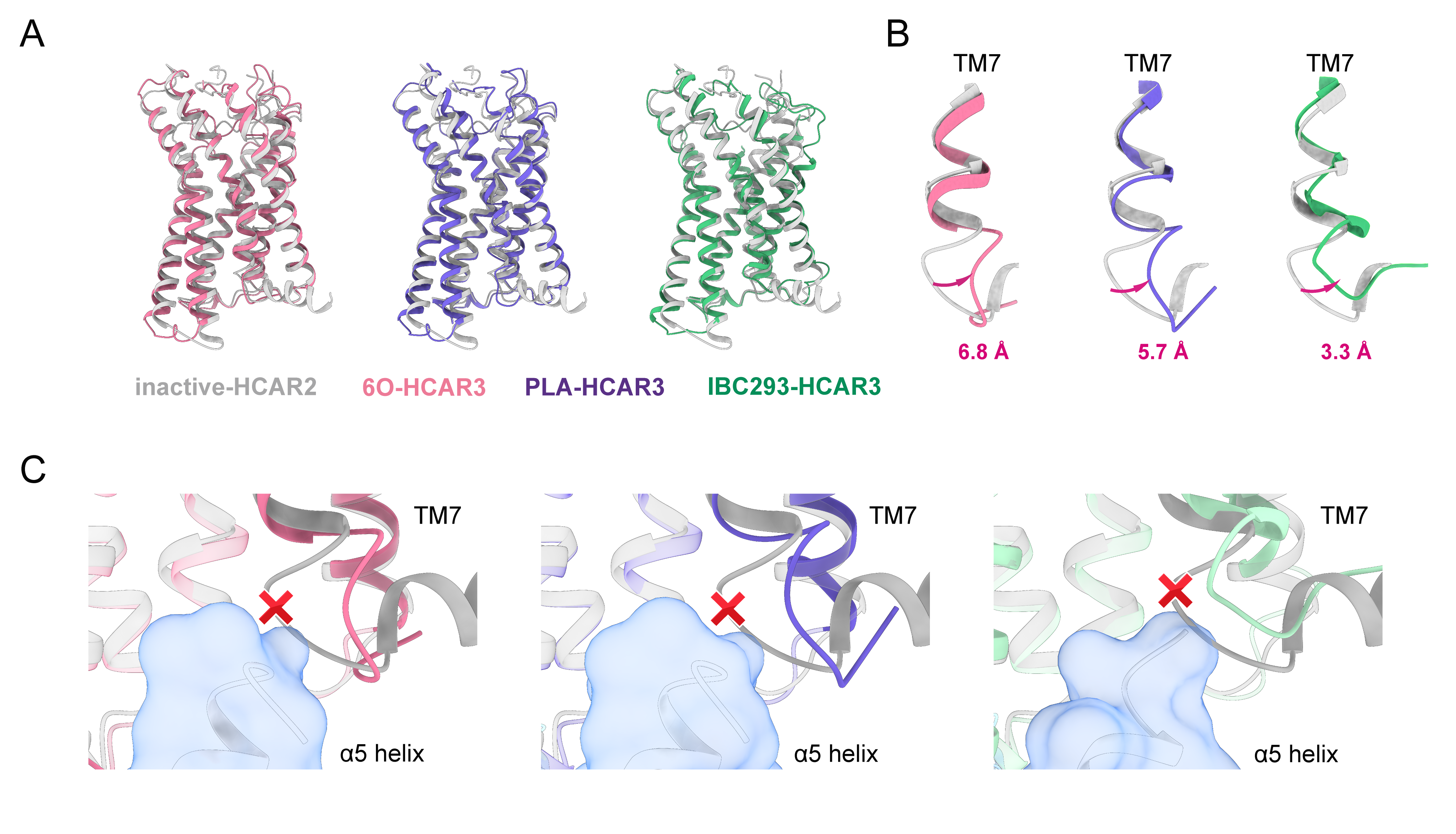

Supplement: S15 Fig — (A) Comparison of 6O-, PLA-, IBC293-bound HCAR3 structures with inactive HCAR2 structure (PDB:7ZL9). (B) Displacements of TM7 upon receptor activation in the structures of 6O-, PLA-, IBC293-bound HCAR3 with inactive HCAR2. (C) Analysis of the mechanism of TM7 interacted with α5 helix of Gi. (TIF) [file pbio.3003480.s015.tif]

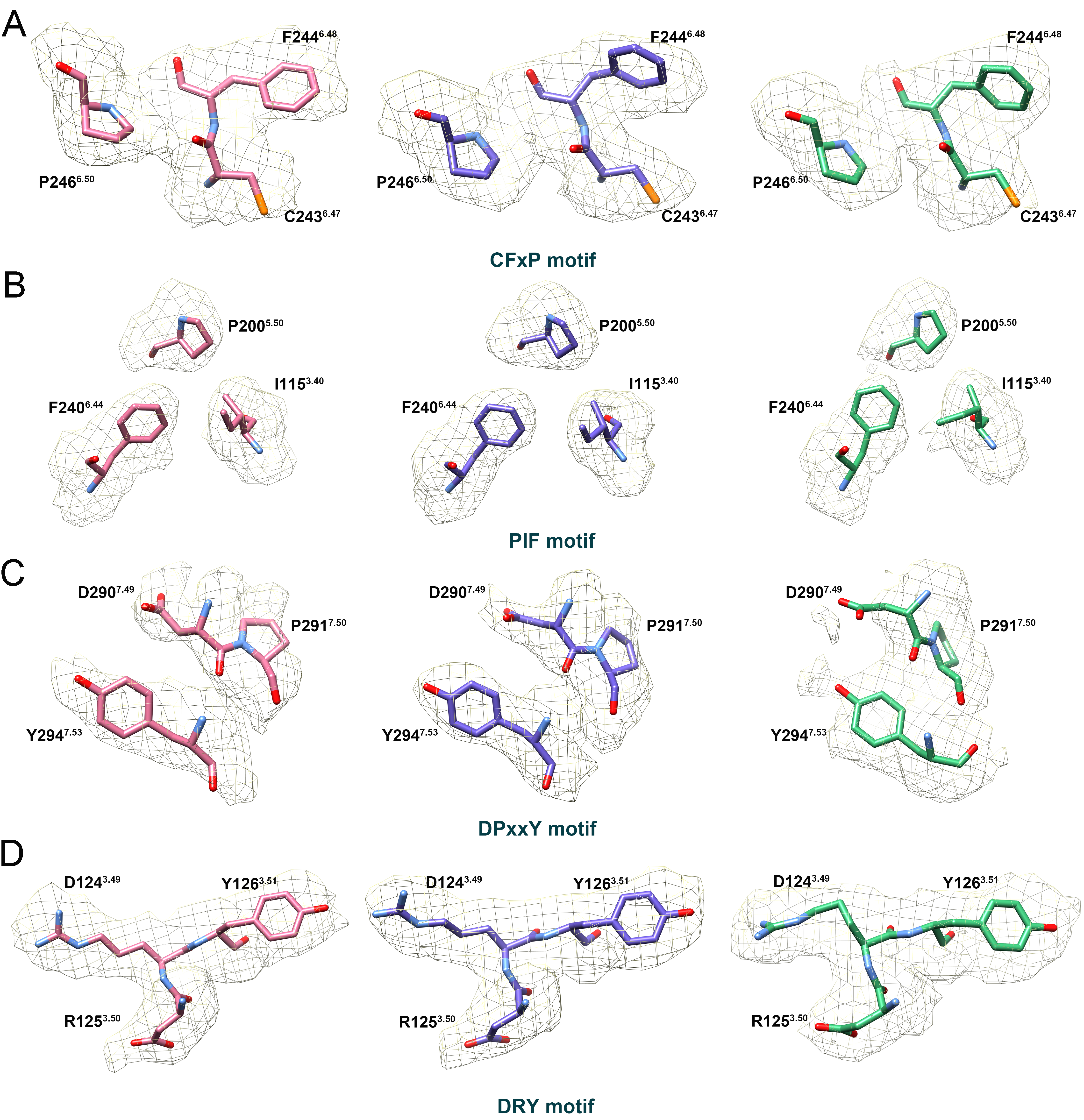

Supplement: S17 Fig — (A) Density of C6.47F6.48xP6.50(CWxP motif in common GPCRs) motif residues. (B) Density of P5.50I3.40F6.44 motif residues. (C) Density of D7.49P7.50xxY7.53 (NPxxY motif in common GPCRs) motif residues. (D) Density of D3.49R3.50Y3.51 motif residues. Pale violet red, 6O-HCAR3; slate blue, PLA-HCAR3; medium sea green, IBC293-HCAR3 (Contour level 6O 4.20 rmsd, PLA 5.60 rmsd, IBC293, 3.90 rmsd). (TIF) [file pbio.3003480.s017.tif]

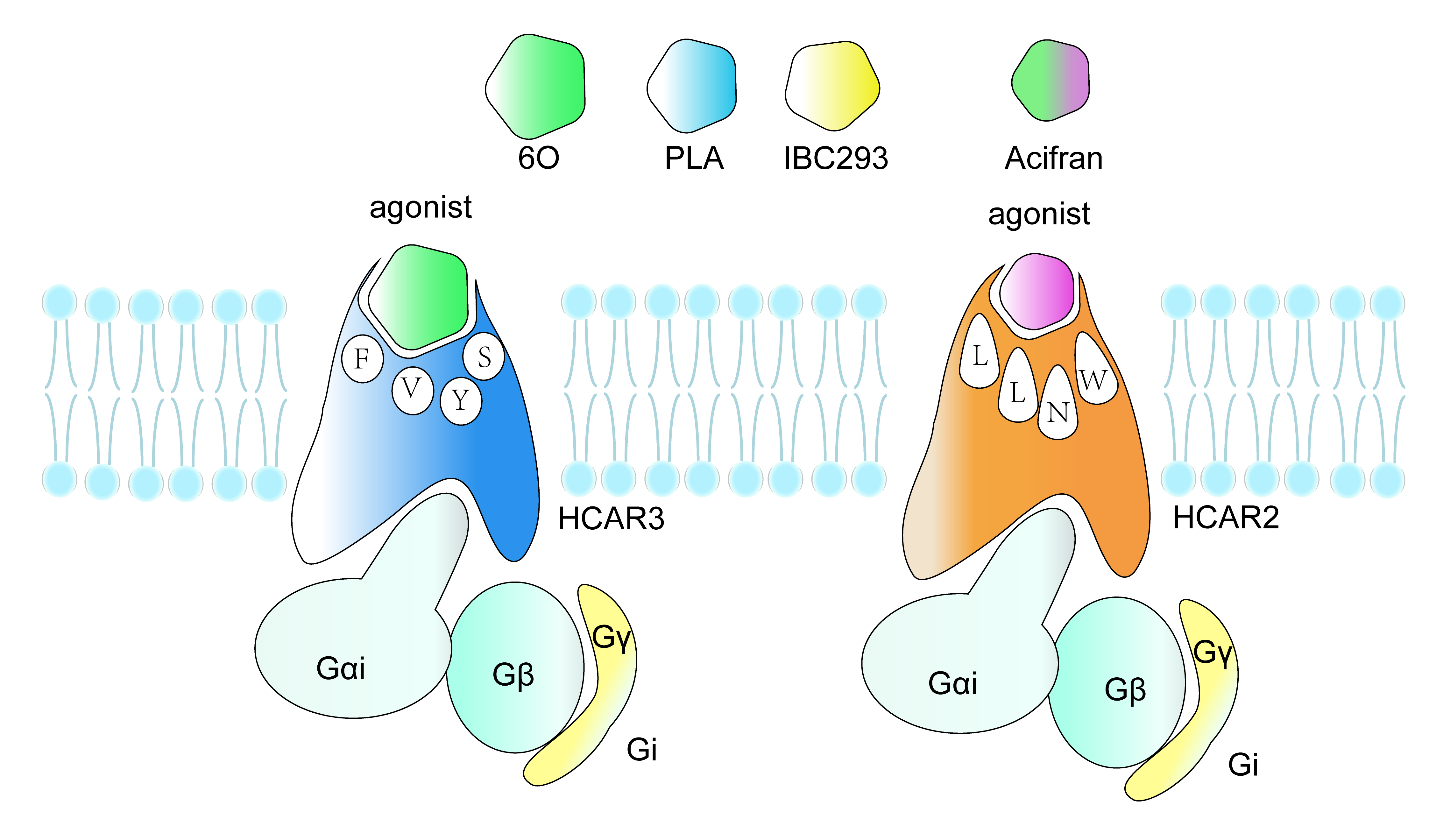

Supplement: S18 Fig — This study primarily elucidates the mechanisms by which the agonists 6O, PLA, and IBC293 bind to HCAR3. 6O is the ligand with the highest binding affinity for HCAR3. 6O, PLA, and IBC293 exhibit strong binding to HCAR3 but weaker binding to HCAR2. The key mechanism lies in the fact that the binding pocket of HCAR3 is larger than that of HCAR2. F/L1073.32, V/L832.60, Y/N862.63, and S/W913.48 are the most critical residues determining the ligand selectivity between HCAR2 and HCAR3. (TIF) [file pbio.3003480.s018.tif]

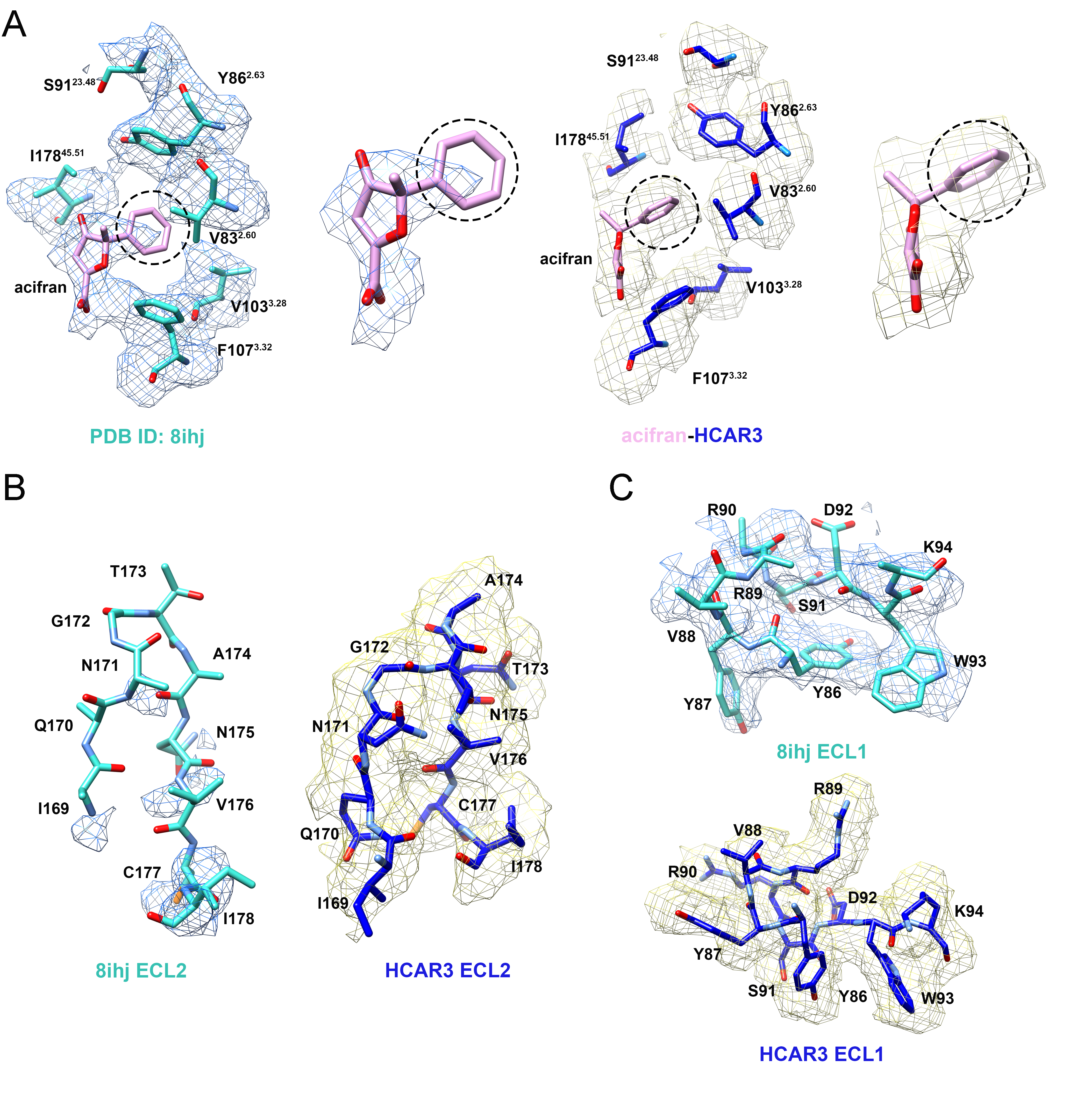

Supplement: S19 Fig — (A) Comparison of electron density for ligand acifran and key residues related to acifran selectivity from Suzuki and colleagues (PDB: 8IHJ) and us. (B, C) Comparison of electron density for ECL1 and ECL2 hairpin regions from Suzuki and colleagues and us. (TIF) [file pbio.3003480.s019.tif]
