# Supplementary material for: Structures of G-protein coupled receptor HCAR3 in complex with selective agonists reveal the basis for ligand recognition and selectivity
Source: PLoS Biol. 2025 Dec 8;23(12):e3003480. doi: 10.1371/journal.pbio.3003480 (PMC12685177; doi:10.1371/journal.pbio.3003480)
Supplement: S1 Table — (PDF) [file pbio.3003480.s020.pdf]

S1 Table. Cryo-EM data collection, refinement and validation statistics.

Data collection and processing	6O-HCAR3	PLA-HCAR3	IBC293 -HCR3	acifran-HCAR3	acifran-HCAR2
Magnification	105,000	105,000	105,000	105,000	105,000
Voltage (kV)	300	300	300	300	300
Electron exposure (e /Å)	45.9	46.4	52.5	53.2	51.3
Defocus range (pm)	-1.0 to -2.0	-1.0 to -2.0	-1.0 to -2.0	-1.0 to -2.0	-1.0 to -2.0
Pixel size (Å)	0.85	0.85	0.83	0.85	0.83
Symmetry imposed	C1	C1	C1	C1	C1
Initial particle images (no.)	2,572,904	3,100,214	2,498,842	2,515,469	3,059,535
Final particle images (no.)	318,073	257,140	268,826	375,522	877,365
Map resolution (Å)	3.31	3.05	3.26	3.18	2.72
FSC threshold	0.143	0.143	0.143	0.143	0.143
**Refinement**					
Initial model used (HCAR3, AlphaFold)	P49019	P49019	P49019	P49019	Q8TDS4
Initial model used (Gi1+scFV16, PDB)	6OMM	6OMM	6OMM	6OMM	6OMM
Map sharpening B-factor (Å^2)	-50	-50	-50	-50	-50
**Model composition**					
Non-hydrogen atoms	8617	8466	7922	8832	8872
Protein residues	1127	1127	1119	1126	1136
**Average B factor (Å^2)**					
Protein	32.52/144.17/65.30	30.00/194.36/82.88	39.60/213.95/96.76	33.52/170.50/81.76	7.46/96.10/40.76
Ligand	110.91/110.91/110.91	97.37/97.37/97.37	147.56/147.56/147.56	89.39/89.39/89.39	61.91/61.91/61.91
**R.m.s. deviations**					

Bond lengths (Å)	0.003	0.002	0.004	0.004	0.003
Bond angles (°)	0.524	0.543	0.615	1.010	0.574
**Validation**					
MolProbity score	1.62	1.47	1.88	2.30	1.71
Clashscore	5.16	4.76	8.08	11.14	8.35
**Ramachandran plot**					
Favored (%)	95.05	96.58	93.18	94.68	96.16
Allowed (%)	4.77	3.33	6.55	5.23	3.66
