# Supplementary material for: Structures of G-protein coupled receptor HCAR3 in complex with selective agonists reveal the basis for ligand recognition and selectivity
Source: PLoS Biol. 2025 Dec 8;23(12):e3003480. doi: 10.1371/journal.pbio.3003480 (PMC12685177; doi:10.1371/journal.pbio.3003480)
Supplement: S2 Table — (PDF) [file pbio.3003480.s021.pdf]

S2 Table. Parameters setting for MD simulation.

		Energy minimization	Equilibration						Production
Ensemble		-	NVT	NVT	NPT	NPT	NPT	NPT	NPT
Time step		-	1 fs	1 fs	1 fs	2 fs	2 fs	2 fs	2 fs
Simulation time		-	125 ps	125 ps	125 ps	500 ps	500 ps	500 ps	500 ns
Thermostat		-	Berendsen	Berendsen	Berendsen	Berendsen	Berendsen	Berendsen	Nose-Hoover
Barstat		-	-	-	Berendsen	Berendsen	Berendsen	Berendsen	Parrinello-Rahman
Temperature		-	310K	310K	310K	310K	310K	310K	310K
Pressure		-	-	-	1 atm	1 atm	1 atm	1 atm	1 atm
Hamonic restrain (kj/mol/nm)	Protein Backbone	4000	4000	2000	1000	500	200	50	-
	Protein sidechain	2000	2000	1000	500	200	50	0	-
	Ligand	4000	4000	2000	1000	500	200	50	-
	Lipid phosphate atom	1000	1000	400	400	200	40	0	-
	Lipid dihedral (C1-C3-C2-O21, C28-C29-C210-C211)	1000	1000	400	200	200	100	0	-
