# Supplementary material for: Structures of G-protein coupled receptor HCAR3 in complex with selective agonists reveal the basis for ligand recognition and selectivity
Source: PLoS Biol. 2025 Dec 8;23(12):e3003480. doi: 10.1371/journal.pbio.3003480 (PMC12685177; doi:10.1371/journal.pbio.3003480)
Supplement: S1 Raw Images — Uncropped Coomassie-stained SDS–PAGE gel used for S1A, S2A, S3A, S4A, and S5A Figs. (PDF) [file pbio.3003480.s022.pdf]

Fig. S1A

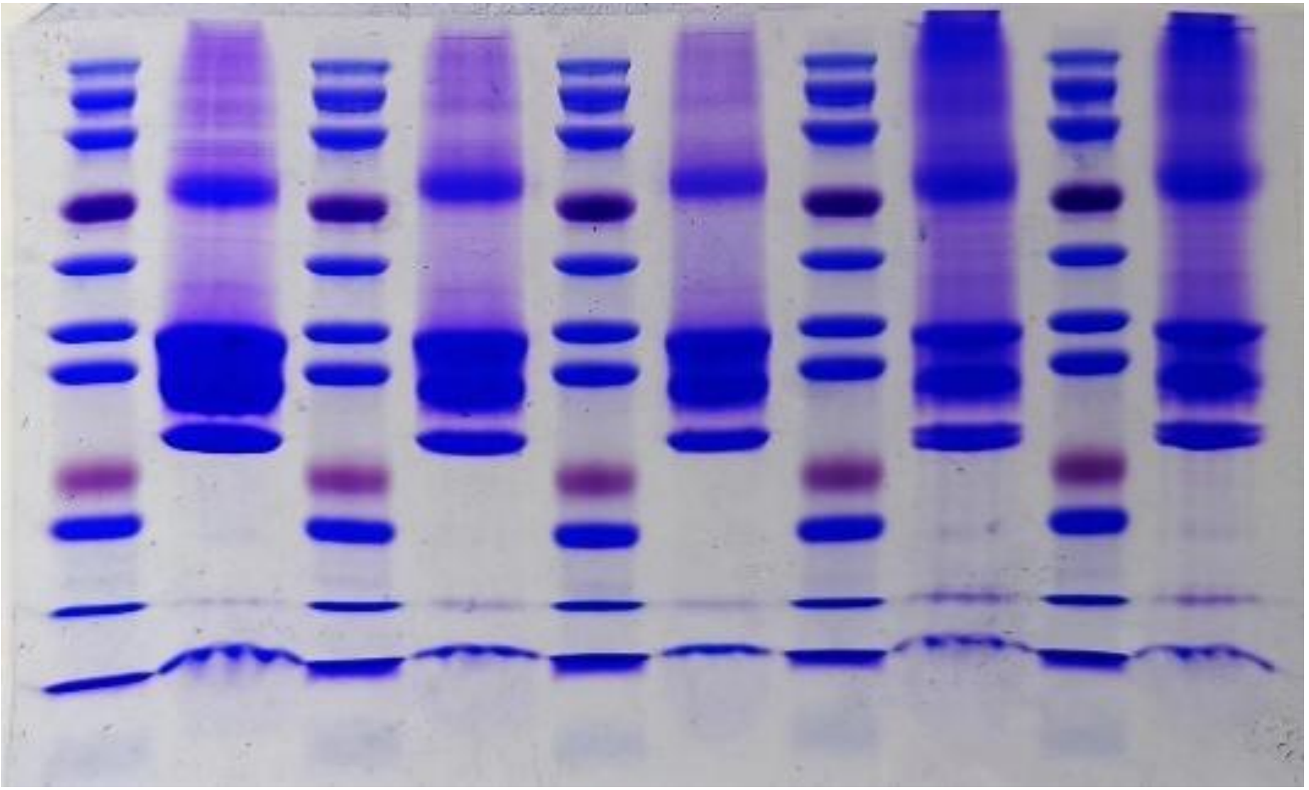

M: Marker
L: Sample loaded

Fig. S2A

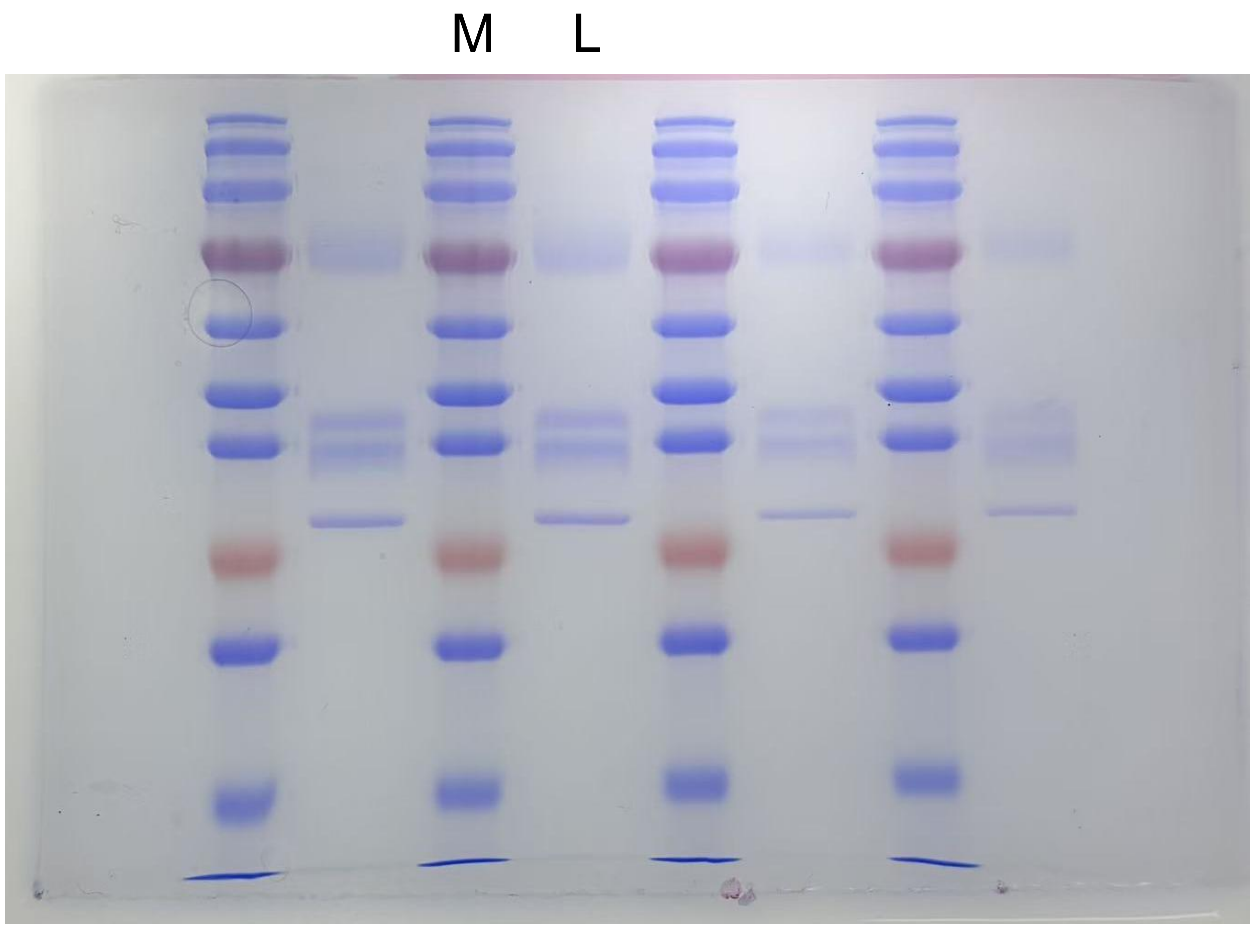

M： Marker
L： Sample loaded

Fig. S3A

M L

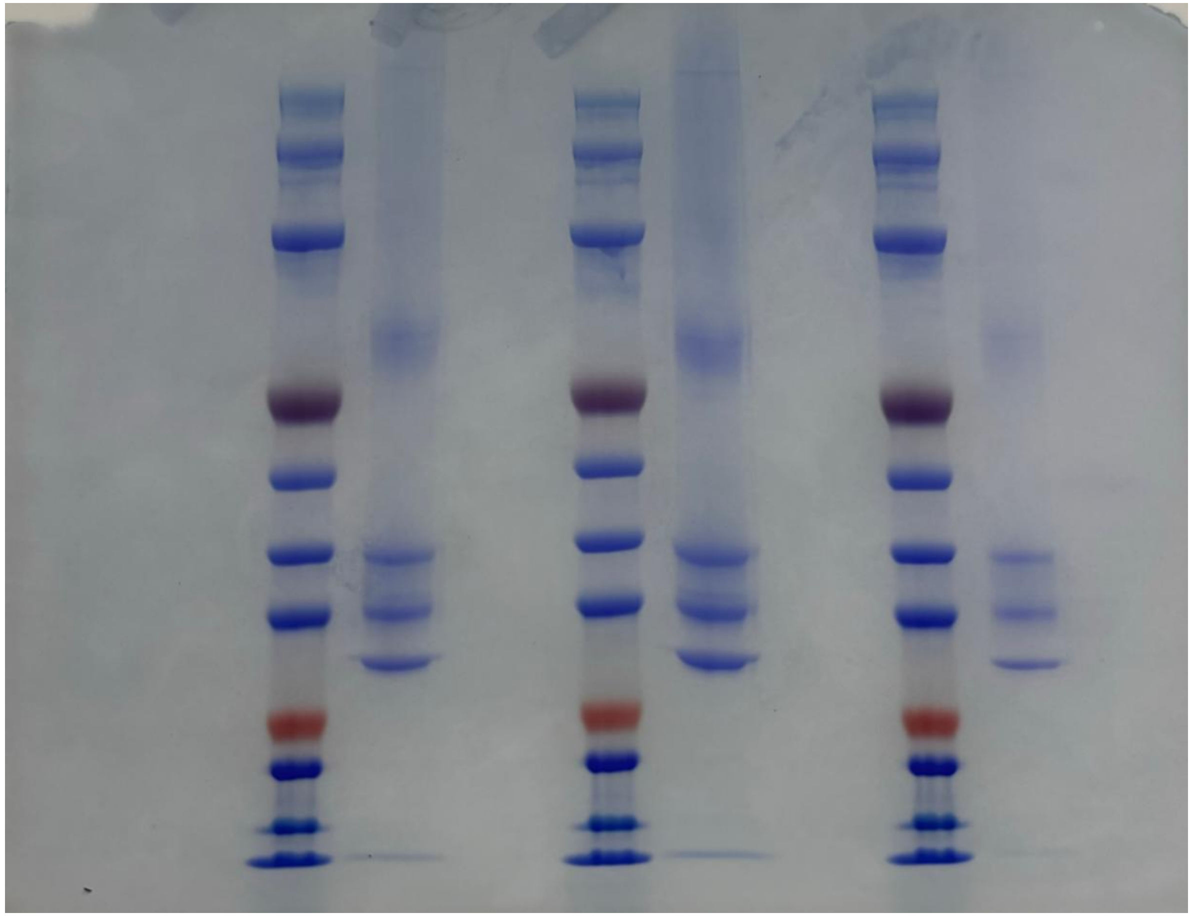

M： Marker
L： Sample loaded

Fig. S4A

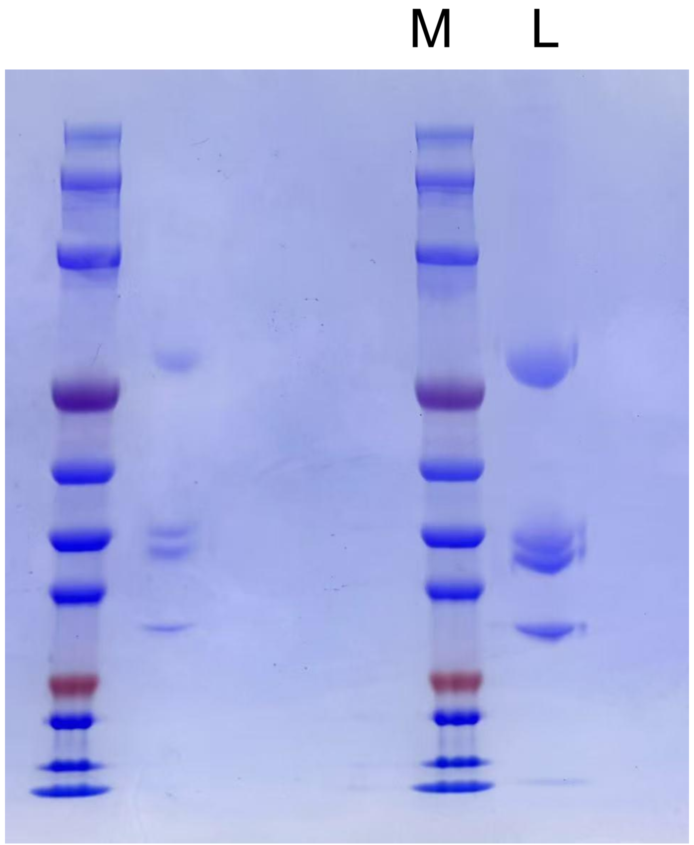

M: Marker
L: Sample loaded

Fig. S5A

M L

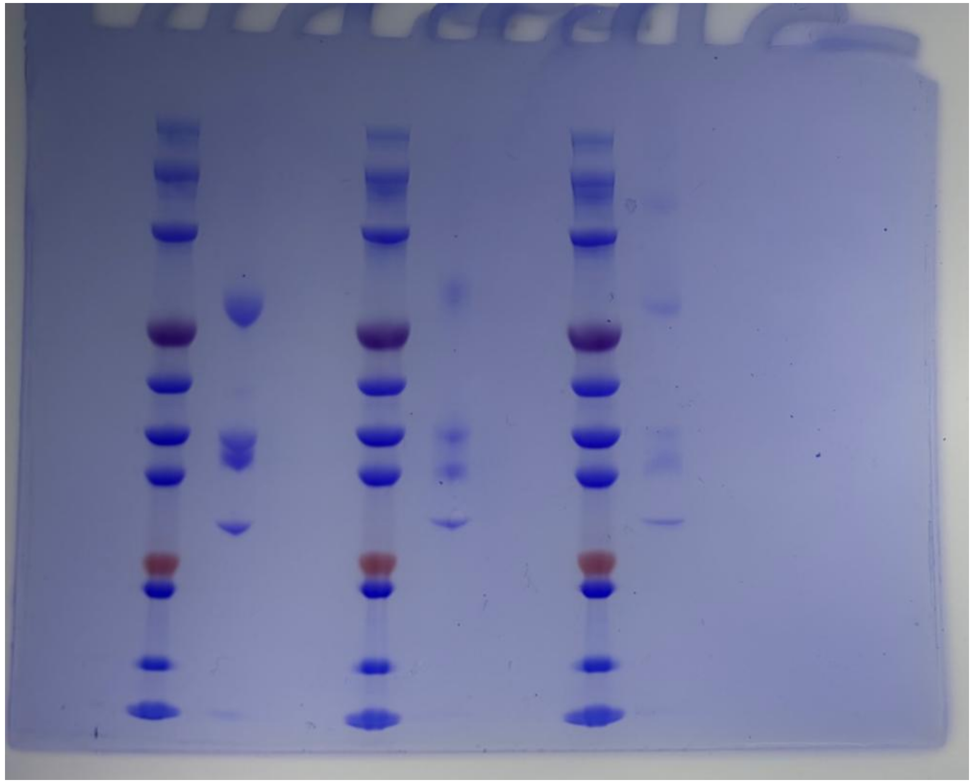

M： Marker
L： Sample loaded
