# Supplementary material for: Structures of G-protein coupled receptor HCAR3 in complex with selective agonists reveal the basis for ligand recognition and selectivity
Source: PLoS Biol. 2025 Dec 8;23(12):e3003480. doi: 10.1371/journal.pbio.3003480 (PMC12685177; doi:10.1371/journal.pbio.3003480)
Supplement: Attachment — Submitted filename: Response to Reviewers.doc [file pbio.3003480.s026.doc]

Dear Editors and Reviewers,

We would like to submit our revision for manuscript PBIOLOGY-D-25-01064R1 entitled “**Structural insights into the ligand selectivity of the human hydroxycarboxylic acid receptor HCAR3 and HCAR2**”. We really appreciate the time both you and the reviewers have taken to detail your helpful comments and suggestions. In this revised version, we have carefully modified our original manuscript according to your comments and suggestions, and have also referred to the regulations and format of your distinguished journal. Below you will find a list of changes for the point-by-point responses to the reviewers’ comments and a list of answers to the questions on the checklist. The corrections were highlighted in yellow in the revised manuscript. We hope that the quality of our manuscript would meet the publication standard of ***PLOS Biology.***

Sincerely,

Yang Du, PhD, associateProfessor/PI

School of Medicine

Kobilka Institute of Innovative Drug Discovery

The Chinese University of Hong Kong,Shenzhen 518172, China

**List of Changes**

**(**Manuscript ID: PBIOLOGY-D-25-01064R1.**)**

**Response to Reviewer 1**

In the article "Structural insights into the ligand selectivity of the human hydroxycarboxylic acid receptor HCAR3 and HCAR2" Ye F, et al solved the cryoEM structures of HCAR3 and HCAR2 bound to a variety of drugs and analyzed the ligand selectivity between two receptors. Their results indicate 1) HCAR3 contains a larger orthosteric binding pocket to accommodate bigger agonists whereas HCAR2 cannot, 2) the flexibility of ECL1 and the presence of F107 in HCAR3 confer its adaptability to ligands with different chemical properties. The conclusion of this study is supported by functional assays including cAMP ELISA and SPR in addition to MD simulations. Overall, this is a carefully designed and succinctly presented report explaining the mechanisms of action for various HCAR3 selective agonists.

**Question 1.** In the introduction, there is no mentioning of previous cryoEM structures of active HCAR2 and HCAR3. A clear description of previous studies and their shortcomings lays solid foundation for unanswered questions that only strengthen the significance of current study. I would like to see the authors acknowledge peers' work.
**Answer 1**. Thank you very much for your kind and professional comments and suggestions. We have added peers’ work in the introduction.

Recently, several cryo-EM structures of HCAR3 bound to ligands 3-hydroxyoctanoic acid, niacin, compound 5c[21], acifran[22], as well as cryo-EM structures of HCAR2 bound to ligands GSK256073, Acipimox[2], MK-1903 and SCH900271[23] have been reported. These works provided valuable insights into ligand recognition and receptor activation mechanism of HCAR3 and HCAR2. However, the structural features and ligand preferences of HCAR3 and HCAR2 required more data support, which could accelerate high-affinity HCAR3 selective drug development. (Please see line 77-83).

**Question 2.** Some figures are misleading or difficult to visualize. For example, Fig.2A presents four ligands at different view angles that causes trouble for this reviewer to compare. Fig.3A and Fig. 4B are so messy that this reviewer still could not distinguish features apart.

**Answer 2**. Thank you very much for your kind suggestions. As shown in the figure below, we have revised Fig. 2A, Fig. 3A and Fig. 4B. (Please see Fig.2A, Fig.3A and Fig. 4B)

(a) Fig. 2A is altered to present ligands at same perspective.

**Fig. 2A** Vertical cross-section of HCAR3 binding pockets in presence of 6O, PLA, IBC293, and acifran.

(b) Pocket color and helix display modes of Fig. 3A are altered to clearly reveal pocket properties.

**Fig. 3A** Surface representation of R1 (large circle) and R2 (small circle) conformations. The structures and agonists are colored differently. Light green and pale violet red, 6O-HCAR3; cyan and slate blue, PLA-HCAR3; khaki and medium sea green, IBC293-HCAR3; plum and dodger blue, acifran-HCAR3.

**(c)** In Fig. 4B, pocket display modes are altered to lines, and HCAR2/HCAR3 pocket color are adjusted to blue/yellow respectively, to clearly reveal pocket size differences.

**Fig. 4B** Surface representation of HCAR2 orthosteric pocket, as well as superimposed HCAR2 and HCAR3 orthosteric pockets. The structures and agonists are colored differently. Medium aquamarine and light salmon, acifran-HCAR2; plum and dodger blue, acifran-HCAR3. Blue, HCAR2 surface; yellow, HCAR3 surface.

**Question 3.** I In the results section, I find "Activation mechanism of HCAR3" is unrelated to the main points of this study. It is also not mentioned in the abstract, introduction, or discussion section of this manuscript. Furthermore, both the active HCAR2 and HCAR3 structures have been reported previously, so this section's novelty is low. Unless further explained, I suggest move this section to supplementary materials so that the article is more focused on ligand selectivity.

**Answer 3.** Thank you very much for your kind suggestions. To emphasize the novelty of the article, we have moved the section of "Activation mechanism of HCAR3" (Fig 5) to supplementary materials. (Please see Figs S15-16.)

**Response to Reviewer 2**

The manuscript "Structural insights into the ligand selectivity of the human hydroxycarboxylic acid receptor HCAR3 and HCAR2" by Ye et al., presents five cryo-EM structures of HCAR3-Gi complexes with agonists compound 6O, PLA, IBC293 and acifran, as well as HCAR2-Gi complex with agonist acifran. The authors clarified the specific binding pocket by examining the binding modes of different ligands in HCAR3 and discussed the subtype selectivity of ligands between HCAR3 and HCAR2. This work provides extensive insights into the ligand selectivity and activation mechanism of HCAR3 and HCAR2, potentially advancing drug development targeting HCAR3.

**Question 1**. The structures of human HCAR3-Gi and HCAR2-Gi bound with the non-selective agonist acifran closely resemble those reported in a 2023 Nature Communications paper (https://doi.org/10.1038/s41467-023-41650-7). Therefore, this section should be significantly shortened. In contrast, the structures of human HCAR3-Gi bound with selective agonists 6O, PLA, and IBC293 represent the novelty and significance of this study.

**Answer 1**. Thank you very much for your kind and professional comments and suggestions. We have

shortened the content of HCAR3-Gi and HCAR2-Gi bound with acifran in Fig 5. Moreover, we have added analysis of the activation mechanism of HCAR3 in the 6O-HCAR3, PLA-HCAR, IBC293-HCAR3 complex.

**(a)** Until now, no inactive HCAR3 structure has been reported. Since HCAR3 and HCAR2 share 96% sequence homology, inactive state HCAR2 structure (PDB ID: 7ZL9) was used to analyze HCAR3 activation mechanism^[29]^. We aligned the structures of 6O-HCAR3, PLA-HCAR3, and IBC293-HCAR3 with the inactive-state HCAR2 structure for comparative analysis (S15A Fig). Relative to inactive HCAR2, these agonist-bound HCAR3 structures exhibited distinct outward displacements in their transmembrane helices: TM7 outward shifted ~6.8 Å (6O-HCAR3), 5.7 Å (PLA-HCAR3), and 3.3 Å (IBC293-HCAR3) (S15B Fig). Notably, the TM7 displacement created a structural niche that accommodated the Gi α5 helix insertion, thereby enabling HCAR3-Gi complex formation, while the absence of this niche in inactive HCAR2 would result in potential steric clashes between TM7 and Gi α5 helix (S15C Fig). (Please see line 210-220, S15 Fig)

**S15 Fig. Helix displacement of ligand bound HCAR3 complexes compared to inactive HCAR2 structure. (A).** Comparison of 6O-，PLA-，IBC293-bound HCAR3 structures with inactive HCAR2 structure (PDB:7zl9). **(B)**. Displacements of TM7 upon receptor activation in the structures of 6O-，PLA-，IBC293-bound HCAR3 with inactive HCAR2. **(C)**. Analysis of the mechanism of TM7 interacted with α5 helix of Gi .

**(b)** Further analysis revealed additional helical rearrangements: In TM5, the extracellular ends displaced ~8.5 Å (6O-HCAR3), 6.7 Å (PLA-HCAR3), and 3.4 Å (IBC293-HCAR3), while intracellular ends shifted ~5.3 Å, 5.1 Å, and 6.0 Å, respectively (S16A Fig). Similarly, TM6 exhibited extracellular displacements of ~2.6 Å, 0.7 Å, and 1.8 Å, paired with intracellular shifts of ~1.9 Å, 1.8 Å, and 0.9 Å (S16B Fig). These concerted movements suggested a global conformational reorganization upon agonist binding.

To investigate the allosteric transmission of activation signal from orthosteric binding pocket towards G protein coupling interface, “CWxP” motif, which is located near ligand binding pocket and contains the “toggle switch” residue, was first analyzed. Interestingly, F244^6.48^ replaced W^6.48^ in HCAR3 and HCAR2, which is common in δ-branch GPCRs (including HCAR family)^[30]^. Compared to inactive HCAR2, F244^6.48^ adopted moderate displacement together with C243^6.47^ and P246^6.50^ from “CWxP” motif (S16C Fig). Similarly, the “PIF” motif, consisting of P200^5.50^, I115^3.40^, and F240^6.44^, underwent moderate residue movement caused by agonist binding (S16D Fig). Notably, moderate conformational changes in “CWxP” and “PIF” motifs, located at the base of orthosteric pocket, “amplified” activation signal and induced larger conformational rearrangements in “NPxxY” and “DRY” motifs, located at G protein interface. In HCAR3 and HCAR2, D290^7.49^ replaced N^7.49^ in “NPxxY” motif. This replacement occurs in 18% of class A GPCRs based on sequence alignment. The difference between aspartic acid and asparagine is probably related to different downstream protein recruitment^[31]^. The other two residues in “NPxxY” motif, P291^7.50^ and Y294^7.53^, rotated along helical axis, resulting in shift of TM7 cytoplasmic end to better accommodate G protein coupling (S16E Fig). Furthermore, large rotameric shift of Y294^7.53^ generated electrostatic interaction with R125^3.50^ from “DRY” motif in 6O and PLA bound HCAR3, breaking ionic lock between D124^3.49^ and R125^3.50^, which stabilized inactive conformation in inactive HCAR2^[32]^. Interestingly, the displacement of Y294^7.53^ and R125^3.50^ was not significant in IBC293 bound HCAR3 (S16F Fig). Holistically, compared to inactive HCAR2, Holistically, compared to inactive HCAR2, key motifs underwent similar displacements in 6O-, PLA-, and IBC293 -bound HCAR3 and HCAR2, with the motif densities being well resolved (S17 Fig), suggesting a typical class A GPCR activation mechanism adopted by HCAR3. These conformational changes opened up the transmembrane domain (TMD) cavity to allow the insertion of α5 helix of Gαi into the TMD core of HCAR3. (Please see line 221-249, S16 Fig)

**S16 Fig. Activation mechanism of HCAR3.** **(A, B)**. Shift of TM5 and TM6 in agonist-bound HCAR3 compared to inactive HCAR2 (PDB ID: 7ZL9). The structures are colored differently: gray, inactive-HCAR2; pale violet red, 6O-HCAR3; slate blue, PLA-HCAR3; medium sea green, IBC293-HCAR3; dodger blue, acifran-HCAR3. **(C-F)**. The key C^6.47^F^6.48^xP^6.50^ (CWxP motif in common GPCRs) **(C)**, P^5.50^I^3.40^F^6.44^ **(D)**, D^7.49^P^7.50^xxY^7.53^ (NPxxY motif in common GPCRs) **(E)**, and D^3.49^R^3.50^Y^3.51^ (**F**) motifs displayed conformational rearrangement in activated HCAR2 and HCAR3.

**Question 2**. The structures were determined by cryo-EM at overall resolutions of 2.7-3.3 Å. According to the local resolution estimations shown in the Supplementary Figures, the resolution in the extracellular region is quite low. Therefore, the cryo-EM densities for the critical residues and extracellular loops (ECLs) involved in ligand binding should be shown.

**Answer 2**. Thank you very much for your kind and professional comments and suggestions. We have added the cryo-EM densities for the critical residues and extracellular loops (ECLs) involved in ligand binding in the supplementary materials. The critical residues and ECL2 associated with ligand binding (6O, S8A Fig; PLA, S8B Fig; IBC293, S8C Fig; acifran-HCAR3, S8D Fig; acifran-HCAR2, S13 Fig) fit well with the cryo-EM densities. (Please see Figs S8 and S13)

**S8 Fig. Electron density of key residues and ECL2 involved in ligand binding.** **(A).** Density of ligand-interacting residues and ECL2 in the 6O complex (Contour level 6O 4.20 rmsd). **(B).** Density of ligand-interacting residues and ECL2 in the PLA complex (Contour level PLA 5.60 rmsd). **(C).** Density of ligand-interacting residues and ECL2 in the IBC293 complex (Contour level 6O 3.90 rmsd). **(D).** Density of ligand-interacting residues and ECL2 in the acifran complex (Contour level acifran 4.70 rmsd).

**S13 Fig. Electron density of key residues and ECL2 involved in Acifran binding to HCAR2. (A).** Electron density of polar and hydrophobic residues in HCAR2 that interact with acifran (Contour level 4.30 rmsd). **(B).** Electron density of ECL2 in HCAR2.

**Question 3**. PLA and IBC293 are relatively small, while the corresponding cryo-EM density are very large. Figure 1C and 1D also suggest that PLA and IBC293 may be modeled in the opposite direction in the HCAR3. How did the authors validate the correct orientation of these ligands?

**Answer 3**. Thank you very much for your kind and professional comments and suggestions. To validate the correct binding poses of PLA and IBC293, we conducted electron density, detailed interaction analysis, and MD simulation for PLA and PLA-opposite pose, as well as IBC293 and IBC293-opposite pose in HCAR3.

**(a)** The orientation of PLA can be determined according to the density map. PLA-opposite pose could not fully match the cryo-EM density due to potential clash with F107^3.32^ (out density region was marked by red box, Figs A-B). Moreover, detailed interaction analysis revealed that PLA-opposite pose lacked hydrogen bonds with R111^3.36^, S179^45.52^, Y284^7.43^ compared to correct pose, which meant a less stable binding pose (Figs C-D).

**Fig. Analysis of the PLA binding orientations. (A-B).** The correct (**A**) and opposite (**B**) poses of PLA. **(C-D).** Detailed interaction analysis of PLA correct pose (**C**) and opposite pose (**D**).

**(b)** The density map for IBC293 is a bit ambiguous. It seems that both the correct pose and opposite pose of IBC293 can fit the density map (Figs A-B). However, the IBC293-opposite pose lacked key polar interaction with Y284^7.43^. Moreover, the isopropyl group formed hydrophobic interactions with V83^2.60^ and W93^23.50^ in the correct pose, while the polar carboxyl group could not in the opposite pose (Figs C-D). The molecular dynamics simulations (MD) also confirmed that the correct pose of IBC293 was more stable. The RMSD of correct pose IBC93 remained around 3.49 Å, but the IBC293-opposite direction was 39.70Å (Figs E-F).

**Fig.** **Analysis of IBC293 binding orientations. (A-B).** The correct (**A**) and opposite (**B**) poses of IBC293. **(C-D).** Detailed interaction analysis of IBC293 correct pose (**C**) and opposite pose (**D**). **(E-F).** MD simulation results of correct pose (**E**) and opposite pose (**F**). RMSD value Raw (light color) and smoothed (10 ns moving average, dark color). Red dash line is the average RMSD value for three replica trajectories.

**Question 4**. Homology mutation experiments and MD simulations indicated that acifran possessed higher stability in HCAR2 pocket than in HCAR3's. However, the authors should also demonstrate the significance of IBC293, 6O, and PLA to HCAR3 relative to HCAR2 through similar homology mutation experiments and MD simulations.

**Answer 4**. Thank you very much for your kind and professional comments and suggestions. We have added results of homology mutation experiments and MD simulations for 6O, IBC293, and PLA to HCAR3 relative to HCAR2, which indicated these ligands possessed higher stability in HCAR3 pocket than in HCAR2's.

**(a)** Mutation of V83^2.60^, Y86^2.63^, S91^3.48^, and F107^3.32^, in HCAR3 to the corresponding amino acids in HCAR2 significantly reduced the binding of 6O to HCAR3 (S14B Fig). Conversely, mutation of L83^2.60^, N86^2.63^, S91^3.48^, and L107^3.32^ in HCAR2 to the HCAR3 residues enhanced the binding of 6O to HCAR2. Similar effects were observed for IBC293 (S14C Fig) and PLA (S14D Fig). (Please see line 199-202, Figs S14 B-D.)

**S14 Fig (B-D). Pocket difference between HCAR3 and HCAR2.** Effects on 6O**(B)**, IBC293**(C)**, PLA**(D)**-induced HCAR3/HCAR2 activation by homology mutation of 83^2.60,^ 86^2.63^, 91^23.48^, and 107^3.32^. The data are presented as means ± SEM. The experiments are performed in triplicates.

**(b)** MD simulations revealed differences in the RMSD of 6O (Fig F), IBC293 (Fig G), and PLA (Fig H) when bound to HCAR3 versus HCAR2, indicating that these three ligands exhibited higher stability in the orthosteric pocket of HCAR3 compared to HCAR2. (Please see line 202-205, Figs 4F-H)

**Fig 4 F-H. Structural basis of ligand selectivity between HCAR3 and HCAR2.** Ligand stability evaluation of 6O **(F)**, IBC293**(G)** and PLA **(H)** in HCAR2 and HCAR3. RMSD value Raw (light color) and smoothed (10 ns moving average, dark color). Red dash line is the average RMSD value for three replica trajectories.

**Question 5**. The angle distribution maps should be included in Supplementary Figures to provide a comprehensive assessment of the final map quality. Additionally, Figure 3A should be clearly labeled for better clarity.

**Answer 5**. Thank you very much for your kind and professional comments and suggestions.

**(a)** The angle distribution maps of 6O-HCAR3, IBC293-HCAR3, PLA-HCAR3, Acifran-HCAR3 and Acifran-HCAR2 were taken from corresponding cryoSPARC refinement outputs, which have been shown in figures (Fig). (Please see S1G-S5G Fig).

**Fig.** Angular distribution calculated in CryoSPARC for the final reconstruction of 6O-HCAR3, IBC293-HCAR3, PLA-HCAR3, Acifran-HCAR3 and Acifran-HCAR2 maps.

**(b)** According to your suggestion, we have relabeled Fig 3A clearer and more legible. (Please see Fig 3A).

**Fig. 3A** Surface representation of R1 (large circle) and R2 (small circle) conformations. The structures and agonists are colored differently. Light green and pale violet red, 6O-HCAR3; cyan and slate blue, PLA-HCAR3; khaki and medium sea green, IBC293-HCAR3; plum and dodger blue, acifran-HCAR3.

**Response to Reviewer 3**

The manuscript by Ye et al describes the structures of two GPCRs, HCAR2 and HCAR3, in complexes with heterotrimeric Gi protein, bound to several agonists: HCAR3-compound 6O, -D-phenyllactate, -IBC293, -acifran, HCAR2-acifran. The authors show that 6O occupies the R1 and R2 positions of the orthosteric binding pocket in HCAR3, explaining the high affinity of the drug. The results also suggest the principles of ligand selectivity between HCAR2 and HCAR3, explained the pi-pi interaction with residue F107 in HCAR3, pocket complementarity (size and specific residues in place).
Overall I think this is a very well crafted manuscript, with high quality illustrations and a consistent story. While some of the structures reported here have previously been published (e.g. HCAR2-acifran, Suzuki et al), the authors argue that their study provides a more complete picture with a better resolved structure. This is a reasonable argument in my opinion.

**Question 1**. My main concern is that the quality of the maps does not allow unambiguous placement of the ligands into the corresponding densities with absolute certainty. This is evident from figure 1B-F. For pretty much each of the compounds, one can propose alternative poses. For 6O, which has a tripod-like structure, potentially at least 3 poses might be possible. For PLA and IBC293, one could imagine rotating the molecules by 180 degrees along two axes, potentially leading to 4 poses per ligand. For acifran in both HCAR2 and HCAR3, two poses might be possible based on the map alone. The authors performed the MD simulations for their chosen poses, but they have not attempted to do the same for the alternative poses of the drugs - the energy of interaction / RMSDs of the compound atoms during the MD simulation would have been robust metrics for selecting the "right" pose. It is of course possible that some logic was applied in placing each of the compounds in a specific orientation pose based on the properties of the binding sites. If so, this should be clarified and ideally still supplemented with MD of the "wrong" poses for good measure.

**Answer 1**. Thank you very much for your kind and professional comments and suggestions. To validate the correct binding poses of 6O, PLA, IBC293, and acifran, we conducted detailed electron density, interaction analysis, and MD simulation for possible three poses of 6O , four poses of PLA, four poses of IBC293, two poses of acifran in HCAR3, and two poses of acifran in HCAR2, respectively.

**(a)** The density map of 6O revealed that pose1 (correct pose), pose2, and pose3 of 6O could fit the density well (Figs A-C). However, compared to 6O-pose1, both 6O-pose2 and 6O-pose3 lacked polar interactions with R111^3.36^, S179^45.52^, Y284^7.43^, which meant these two alternated poses lacked stability within the ligand-binding pocket (Figs D-F). The molecular dynamics simulations (MD) also confirmed that the correct 6O-pose1 was more stable with an average RMSD of 2.46Å (Figs G). In contrast, 6O-pose2 and 6O-pose3 exhibited higher average RMSD values of 6.84 Å and 5.85 Å, respectively (Figs H-I). Both interaction analyses and MD consistently revealed that the current 6O-pose1 was a more stable binding pose.

**Fig. Analysis of 6O binding orientations. (A-C).** The pose1 (correct pose, **A**), pose2 (**B**), pose3 (**C**) of 6O. **(D-F).** Detailed interaction analysis of 6O-pose1 (**D**), 6O-pose2 (**E**), 6O-pose3 (**F**). **(G-I).** MD simulation results of 6O-pose1 (**G**), 6O-pose2 (**H**), 6O-pose3 (**I**). RMSD value Raw (light color) and smoothed (10 ns moving average, dark color). Red dash line is the average RMSD value for three replica trajectories.

**(b)** The density map of PLA revealed that pose1 (correct pose) and pose2 of PLA could fit the density well (Figs A-B), while both PLA-pose3 and PLA-pose4 had out density regions (marked by red boxes) due to potential clash with F107^3.32^ (Figs C-D). Detailed interaction analysis revealed that, compare to PLA-pose1, PLA-pose2 lacked the hydrogen bond with S179^45.52^, meanwhile both PLA-pose3 and PLA-pose4 lacked key polar interactions with R111^3.36^, S179^45.52^, Y284^7.43^ (Figs E-H). The density map and interaction analysis demonstrated that the PLA-pose3 and PLA-pose4 were incorrect binding conformations. Moreover, MD simulations revealed that the average RMSD of PLA-pose1 exhibited lower values than that of PLA-pose2, which indicated that PLA-pose1 was a more stable binding pose. (Figs I-J).

**Fig. Analysis of PLA binding orientations. (A-D).** The pose1 (correct pose, **A**), pose2 (**B**), pose3 (**C**), pose4 (**D**) of PLA. **(E-H).** Detailed interaction analysis of PLA-pose1 (**E**), PLA-pose2 (**F**), PLA-pose3 (**G**), PLA-pose4 (**H**). **(I-J).** MD simulation results of PLA-pose1 (**I**), PLA-pose2 (**J**). RMSD value Raw (light color) and smoothed (10 ns moving average, dark color). Red dash line is the average RMSD value for three replica trajectories.

**(c)** The density map of IBC293 demonstrated that pose1 (correct pose), pose2, pose3, and pose4 of IBC293 could fit the density well (Figs A-D). However, compared to IBC293-pose1, detailed interaction analysis revealed that the IBC293-pose2 lacks hydrophobic interactions with residues V83^2.60^, and W93^23.50^, while IBC293-pose3 and IBC293-pose4 lacked key polar interactions with Y284^7.43^ (Figs E-H). MD results also demonstrated that the average RMSD of IBC293-pose1 (3.49 Å) was significantly lower than those of IBC293-pose2 (8.11 Å), IBC293-pose3 (11.54 Å), and IBC293-pose4 (39.70 Å) (Figs. I-L). Both interaction analyses and MD simulations consistently indicated that the current IBC293-pose1 adopted the most stable binding conformation.

**Fig. Analysis of IBC293 binding orientations. (A-D).** The pose1 (correct pose, **A**), pose2 (**B**), pose3 (**C**), pose4 (**D**) of IBC293. **(E-H).** Detailed interaction analysis of IBC293-pose1 (**E**), IBC293-pose2 (**F**), IBC293-pose3 (**G**), IBC293-pose4 (**H**). **(I-L).** MD simulation results of IBC293-pose1 (**I**), IBC293-pose2 (**J**), IBC293-pose3 (**K**), IBC293-pose4 (**L**). RMSD value Raw (light color) and smoothed (10 ns moving average, dark color). Red dash line is the average RMSD value for three replica trajectories.

**(d)** For acifran-HCAR3, alternated pose (acifran-HCAR3-pose2) could fit density well (Figs A-B). However, detailed interaction analysis revealed that the key polar interactions with R111^3.36^, S179^45.52^, Y284^7.43^, which were found in correct acifran-HCAR3-pose1, were absent in the alternated acifran-HCAR3-pose2. Moreover, acifran-HCAR3-pose2 lacked key pi-pi stacking with F107^3.32^ compared to acifran-HCAR3-pose1 (Figs C-D). Consistently, high average RMSD value of MD simulation results for acifran-HCAR3-pose2 (RMSD 6.75Å) also suggested that the alternated pose was less stable in the ligand binding pocket than correct acifran-HCAR3-pose1 (RMSD 3.68Å) (Figs I-J). Additionally, in the acifran–HCAR3 structure previously reported by Suzuki et al., the carboxyl group of acifran was oriented toward the intracellular side, further supporting the correctness of our current acifran-HCAR3-pose1 orientation.

**Fig. Analysis of acifran-HCAR3 binding orientations. (A-B).** Electron density of acifran-HCAR3-pose1 (correct pose, **A**) and acifran-HCAR3-pose2 (**B**). **(C-D).** Detailed interaction analysis of acifran-HCAR3-pose1 (**C**) and acifran-HCAR3-pose2 (**D**). **(E-F).** MD simulation results of acifran-HCAR3-pose1 and acifran-HCAR3-pose2. RMSD value Raw (light color) and smoothed (10 ns moving average, dark color). Red dash line is the average RMSD value for three replica trajectories.

**(e)** For acifran-HCAR2, alternated poses (acifran-HCAR2-pose2) could fit density well (Figs A-B). However, compared to correct acifran-HCAR2-pose1, detailed interaction analysis revealed that the polar interactions with R111^3.36^, S179^45.52^, Y284^7.43^ were absent in acifran-HCAR2-pose2 (Figs C-D). Consistently, high average RMSD value of MD simulation results for acifran-HCAR2-pose2 (RMSD 6.46Å) also suggested that the alternated pose was less stable in the ligand binding pocket than correct acifran-HCAR2-pose1 (RMSD 2.57Å) (Figs E-F). Additionally, in the acifran–HCAR2 structures previously reported by Suzuki et al., the carboxyl group of acifran was oriented toward the intracellular side, further supporting the correctness of our current acifran-HCAR1-pose1 orientation.

**Fig. Analysis of acifran-HCAR2 binding orientations. (A-B).** Electron density of acifran-HCAR2-pose1 (correct pose, **A**) and acifran-HCAR2-pose2 (**B**). **(C-D).** Detailed interaction analysis of acifran-HCAR2-pose1 (**C**) and acifran-HCAR2-pose2 (**D**). **(E-F).** MD simulation results of acifran-HCAR2-pose1 and acifran-HCAR2-pose2. RMSD value Raw (light color) and smoothed (10 ns moving average, dark color). Red dash line is the average RMSD value for three replica trajectories.

**Question 2**. Map to model FSC curves are missing for all structures. For completeness, please provide those.

**Answer 2**. Thank you very much for your kind and professional comments and suggestions. We have added the map to model FSC curves in the Supplementary materials (S6. Fig)**.** (Please see line 102-103, Fig. S6)

**S6 Fig. Map to model FSC curves. (A).** Map to model FSC curve of 6O-HCAR3 complex. **(B).** Map to model FSC curve of PLA-HCAR3 complex. **(C).** Map to model FSC curve of IBC293-HCAR3 complex. **(D).** Map to model FSC curve of Acifran-HCAR3 complex. **(E).** Map to model FSC curve of Acifran-HCAR2 complex.

**Question 3**. Fig 4B right panel and Supplementary Fig 11A right panel are somewhat confusing, with multiple elements placed on top of each other - I am sure this figure can be simplified. Maybe it is easier to digest this material without the complex alignment of the surfaces - just the ligands. Or perhaps showing the structures side by side would simplify the presentation. In the same figure panels, and also in Fig 3A the ripple effect on the surfaces of the helices is visually slightly distracting. This is really a minor point and a matter of taste.

**Answer 3**. Thank you very much for your kind and professional comments and suggestions. We adjusted the pocket surface color and helix display modes for better distinguishability of pocket size difference. (Please see Figs 3A, 4B, and S14A).

(a) Pocket color and helix display modes of Fig 3A are altered to clearly reveal pocket properties.

**Fig. 3A** Surface representation of R1 (large circle) and R2 (small circle) conformations. The structures and agonists are colored differently. Light green and pale violet red, 6O-HCAR3; cyan and slate blue, PLA-HCAR3; khaki and medium sea green, IBC293-HCAR3; plum and dodger blue, acifran-HCAR3.

**(b)** In Fig 4B, pocket display modes are altered to lines, and HCAR2/HCAR3 pocket color are adjusted to blue/yellow respectively, to clearly reveal pocket size differences.

**Fig. 4B** Surface representation of HCAR2 orthosteric pocket, as well as superimposed HCAR2 and HCAR3 orthosteric pockets. The structures and agonists are colored differently. Medium aquamarine and light salmon, acifran-HCAR2; plum and dodger blue, acifran-HCAR3. Blue, HCAR2 surface; yellow, HCAR3 surface.

**(c)** In Fig S11A (modified to **S14 Fig**), pocket display modes are altered to lines, and HCAR3/HCAR2 pocket color are adjusted to yellow/blue respectively, to clearly reveal R2, which was occupied by a thiophene group of 6O, was absent in HCAR2 orthosteric pocket..

**S14 Fig. (A)** Surface representation of HCAR3 and HCAR2 orthosteric pocket, as well as superimposed HCAR2 and HCAR3 orthosteric pockets. The structures and agonists are colored differently. Medium aquamarine and light salmon, acifran-HCAR2; light green and pale violet red, 6O-HCAR3. Blue, HCAR2 surface; yellow, HCAR3 surface.

**Question 4**. A summary figure that outlines the key findings in a sketch would be very useful.

**Answer 4**. Thank you very much for your kind and professional comments and suggestions. We have added an illustration to outline the main messages of this Manuscript in Fig 5.

**(a)** Our study mainly elucidates the cryo-EM structures of agonist (6O, PLA, IBC293)-bound HCAR3 complexes, along with the selective mechanisms between HCAR3 and HCAR2**.** The cryo-EM structures reveal the occupation to two hydrophobic regions R1 and R2 of HCAR3's orthosteric pocket constitutes the primary determinant for 6O's high-affinity binding to HCAR3. We have also elucidated the mechanism underlying the distinctive binding pose of IBC293 to HCAR3，unlike other HCAR3 ligands.

**(b)** Regarding the mechanism of ligands recognition, our results elucidated the mechanism of the inability of HCAR2 to recognize HCAR3 specific agonists compound 6O, PLA, and IBC293. F/L107^3.32^ are the most critical residue determine the ligand selectivity between HCAR2 and HCAR3, and V/L83^2.60^, Y/N86^2.63^, S/W91^23.48^ determine the pocket size divergence between these two receptors. (Please see line 255-261, Fig. 5)

**Fig 5.** **Mechanism diagram of ligand recognition and selectivity of HCAR3 and HCAR2.** This study primarily elucidates the mechanisms by which the agonists 6O, PLA, and IBC293 bind to HCAR3. 6O is the ligand with the highest binding affinity for HCAR3. 6O, PLA, and IBC293 exhibit strong binding to HCAR3 but weaker binding to HCAR2. The key mechanism lies in the fact that the binding pocket of HCAR3 is larger than that of HCAR2. F/L107^3.32^, V/L83^2.60^, Y/N86^2.63^, and S/W91^3.48^ are the most critical residues determining the ligand selectivity between HCAR2 and HCAR3.
