# Supplementary material for: Structures of G-protein coupled receptor HCAR3 in complex with selective agonists reveal the basis for ligand recognition and selectivity
Source: PLoS Biol. 2025 Dec 8;23(12):e3003480. doi: 10.1371/journal.pbio.3003480 (PMC12685177; doi:10.1371/journal.pbio.3003480)
Supplement: Attachment — Submitted filename: Cover Letter (Response to Editors).docx [file pbio.3003480.s027.docx]

Dear Editors,

We would like to submit our revision for manuscript PBIOLOGY-D-25-01064R2 entitled “**Structures of G-protein coupled receptor HCAR3 in complex with selective agonists reveal the basis for ligand recognition and selectivity**”. We really appreciate the time both you and the reviewers have taken to detail your helpful comments and suggestions. In this revised version, we have carefully modified our original manuscript according to your comments and suggestions, and have also referred to the regulations and format of your distinguished journal. Below you will find a list of changes for the point-by-point responses to the reviewers’ comments and a list of answers to the questions on the checklist. The corrections were highlighted in yellow in the revised manuscript. We hope that the quality of our manuscript would meet the publication standard of ***PLOS Biology.***

Sincerely,

Yang Du, PhD, associateProfessor/PI

School of Medicine

Kobilka Institute of Innovative Drug Discovery

The Chinese University of Hong Kong,Shenzhen 518172, China

**List of Changes**

**(**Manuscript ID: PBIOLOGY-D-25-01064R2 **)**

**Response to Editors**

**Question A.** Please note that we considering your manuscript as a Short Report, which has a maximum of 4 main figures. Therefore, we ask that the number of main figures is reduced at this stage by either combining main figures or moving one of the figures to the Supplementary (Figure 5?).
**Answer A**. Thank you very much for your kind and professional comments and suggestions. The revised-manuscript is 4 main figures. We have moved the Figure 5 to the supplementary materials. (Please see line 257, S18 Fig.).

**Question B.** We routinely suggest changes to titles to ensure maximum accessibility for a broad, non-specialist readership. In this case, we would suggest a minor edit to the title, as follows. Please ensure you change both the manuscript file and the online submission system, as they need to match for final acceptance:

“Structures of G-protein coupled receptor HCAR3 in complex with selective agonists reveal the basis for ligand recognition and selectivity”

**Answer B**. Thank you very much for your kind suggestions. As shown in the revised-manuscript, we have revised the title to “Structures of G-protein coupled receptor HCAR3 in complex with selective agonists reveal the basis for ligand recognition and selectivity”. (Please see the revised-manuscript and the online submission system)

**Question C.** Thank you for providing the structural data in the PDB and EMDB databases. However, we note that the data is currently on hold for release. We ask that you please make the structures publicly available at this stage before publication.

**Answer C.** Thank you very much for your kind suggestions. According to your requirement, we have contacted the PDB databases and made the structural data publicly available soon.

**Question D**. Please also ensure that each of the relevant figure legends in your manuscript include information on *WHERE THE UNDERLYING DATA CAN BE FOUND*, and ensure your supplemental data file/s has a legend.

**Answer D**. Thank you very much for your kind suggestions. All figure legends have been included in our manuscript.

Fig 1-Fig 4: Please see line 529-563

S1 Fig-S19 Fig, S1 Table, S2 Table, S1 Raw Images, S1 Data: Please see line 754-923

**Question E**. Per journal policy, if you have generated any custom code during the course of this investigation, please make it available without restrictions. Please ensure that the code is sufficiently well documented and reusable, and that your Data Statement in the Editorial Manager submission system accurately describes where your code can be found.

Please note that we cannot accept sole deposition of code in GitHub, as this could be changed after publication. However, you can archive this version of your publicly available GitHub code to Zenodo. Once you do this, it will generate a DOI number, which you will need to provide in the Data Accessibility Statement (you are welcome to also provide the GitHub access information). See the process for doing this here: https://apc01.safelinks.protection.outlook.com/?url=https%3A%2F%2Fdocs.github.com%2Fen%2Frepositories%2Farchiving-a-github-repository%2Freferencing-and-citing-content&data=05%7C02%7Cyangdu%40cuhk.edu.cn%7Cd7b7b746459e450dc33808de076b6e80%7Ce5898b7a9c874a4bb22a2df3f355e01e%7C0%7C0%7C638956355177791583%7CUnknown%7CTWFpbGZsb3d8eyJFbXB0eU1hcGkiOnRydWUsIlYiOiIwLjAuMDAwMCIsIlAiOiJXaW4zMiIsIkFOIjoiTWFpbCIsIldUIjoyfQ%3D%3D%7C0%7C%7C%7C&sdata=hJFvzXxyLALuqhnfQOYaQufvn95XRsS902r%2BqCHQHcw%3D&reserved=0

**Answer E**. No custom code is generated during the course.

**Question F**. Please note that per journal policy, the model system/species studied should be clearly stated in the abstract of your manuscript.

**Answer F**. Thank you very much for your important reminder. We have now revised the abstract to clearly state the model system used in our study. Specifically, we have added “we used Sf9 cells to express HCAR3-Gi and HCAR2-Gi complexes” and “combined with cAMP assay in 293 HEK-293 cells” in the abstract. (Please see line 29-30 and line 34)

**Question G**. As you address these items, please take this last chance to review your reference list to ensure that it is complete and correct. If you have cited papers that have been retracted, please include the rationale for doing so in the manuscript text, or remove these references and replace them with relevant current references. Any changes to the reference list should be mentioned in the cover letter that accompanies your revised manuscript.

**Answer G**. Thank you very much for your kind suggestion. We have checked the reference list and confirmed that no retracted papers are cited. All the reference formats have been rechecked and revised to ensure that they are complete and correct. (Please see line 565-737)
